# Sequencing of DISC1 Pathway Genes Reveals Increased Burden of Rare Missense Variants in Schizophrenia Patients from a Northern Swedish Population

**DOI:** 10.1371/journal.pone.0023450

**Published:** 2011-08-11

**Authors:** Lotte N. Moens, Peter De Rijk, Joke Reumers, Maarten J. A. Van Den Bossche, Wim Glassee, Sonia De Zutter, An-Sofie Lenaerts, Annelie Nordin, Lars-Göran Nilsson, Ignacio Medina Castello, Karl-Fredrik Norrback, Dirk Goossens, Kristel Van Steen, Rolf Adolfsson, Jurgen Del-Favero

**Affiliations:** 1 Applied Molecular Genomics Group, Department of Molecular Genetics, Flanders Institute for Biotechnology (VIB), Flanders, Belgium; 2 University of Antwerp (UA), Antwerp, Belgium; 3 SWITCH Laboratory, Flanders Institute for Biotechnology (VIB), Flanders, Belgium; 4 Vrije Universiteit Brussel (VUB), Brussels, Belgium; 5 Division of Psychiatry, Department of Clinical Sciences, Umeå University, Umeå, Sweden; 6 Department of Psychology, Stockholm University, Stockholm, Sweden; 7 Functional Genomics Unit, Bioinformatics and Genomics Department, Prince Felipe Research Centre (CIPF), Valencia, Spain; 8 Systems and Modeling Unit, Montefiore Institute/GIGA, University of Liège, Liège, Belgium; VU University Medical Center and Center for Neurogenomics and Cognitive Research, Netherlands

## Abstract

In recent years, *DISC1* has emerged as one of the most credible and best supported candidate genes for schizophrenia and related neuropsychiatric disorders. Furthermore, increasing evidence – both genetic and functional – indicates that many of its protein interaction partners are also involved in the development of these diseases. In this study, we applied a pooled sample 454 sequencing strategy, to explore the contribution of genetic variation in DISC1 and 10 of its interaction partners (ATF5, Grb2, FEZ1, LIS-1, PDE4B, NDE1, NDEL1, TRAF3IP1, YWHAE, and ZNF365) to schizophrenia susceptibility in an isolated northern Swedish population. Mutation burden analysis of the identified variants in a population of 486 SZ patients and 514 control individuals, revealed that non-synonymous rare variants with a MAF<0.01 were significantly more present in patients compared to controls (8.64% versus 4.7%, P = 0.018), providing further evidence for the involvement of *DISC1* and some of its interaction partners in psychiatric disorders. This increased burden of rare missense variants was even more striking in a subgroup of early onset patients (12.9% versus 4.7%, P = 0.0004), highlighting the importance of studying subgroups of patients and identifying endophenotypes. Upon investigation of the potential functional effects associated with the identified missense variants, we found that ∼90% of these variants reside in intrinsically disordered protein regions. The observed increase in mutation burden in patients provides further support for the role of the DISC1 pathway in schizophrenia. Furthermore, this study presents the first evidence supporting the involvement of mutations within intrinsically disordered protein regions in the pathogenesis of psychiatric disorders. As many important biological functions depend directly on the disordered state, alteration of this disorder in key pathways may represent an intriguing new disease mechanism for schizophrenia and related neuropsychiatric diseases. Further research into this unexplored domain will be required to elucidate the role of the identified variants in schizophrenia etiology.

## Introduction

Schizophrenia (SZ) is a severe psychiatric disorder affecting ∼1% of the population worldwide. The disease is characterized by *positive symptoms*, including hallucinations, delusions and disturbances in thoughts, as well as *negative symptoms* such as lack of motivation and attention, asocial behavior and cognitive dysfunction. With a heritability of ∼60–80% [1], SZ has a clear genetic component, but despite major efforts during the last decades to identify genetic risk factors, only a handful of candidate genes could be replicated independently [2]–[4] and even a smaller number demonstrated clear biological support.

One recent and promising exception is *Disrupted in Schizophrenia 1* (*DISC1*), which was originally identified via a balanced t(1;11) chromosomal translocation segregating with a wide spectrum of psychiatric disorders in a large Scottish pedigree [5]. Since its discovery, several independent linkage and association studies in diverse populations and various phenotype models – including SZ, bipolar disorder, major depression, as well as various neurophysiological, cognitive and structural traits – have confirmed the original findings, further supporting a central role for *DISC1* genetic variation in conferring susceptibility to psychiatric illness [6]–[27] .

Nevertheless, except for the translocation no specific causal variant has yet been identified, , and most compelling evidence so far has come from functional genomic and cell biological analyses, suggesting an essential role of DISC1 in neuronal development, including adult neurogenesis and signaling [28]–[36].

Research into DISC1's multiple interaction partners has proved very valuable in elucidating its biological functions, further implicating it in essential processes of brain development and adult neuronal function [29], [33], [37]–[39]. With over 15 *in vivo* confirmed protein interactors [33], [37]–[46], and many more potential interactions (>60, identified by yeast two-hybrid screens) [43], [47]–[48], DISC1 is considered a central ‘hub’ protein, connecting numerous functional systems, including neuronal migration, neurite outgrowth, cytoskeletal modulation and cAMP signaling, within the brain [36], [47]. The observation that several of these interaction partners have been identified as independent (genetic) susceptibility factors for neuropsychiatric diseases [49], and the potential crosstalk with known schizophrenia risk factors such as dysbindin and neuregulin, which share multiple putative binding partners with DISC1 [47], indicate that not just DISC1, but a multidimensional ‘DISC1 pathway’ is involved in the etiology of psychiatric diseases.

At present, however, little is known on the role of genetic variation in the individual DISC1 interactors, or combinations of the different interaction partners, in the development of these disorders. Therefore, detailed studies of the various components of the DISC1 pathway will be essential to fully understand the role of this complex molecular network in conferring susceptibility to psychiatric diseases. Studying a set of candidate genes in convergent molecular risk networks – like the DISC1 pathway – is an attractive strategy, as it allows to investigate the cumulative effects of (moderately deleterious) mutations that, because of their molecular connection, could cause genetic impairment of pathway activity and thereby lead to phenotypic effects. Hereby, it is of particular importance to search for rare mutations (with a low population frequency (<1%), and a relatively high penetrance), as it has been suggested that these variants, or combinations thereof, could explain a substantial fraction of common disorders like SZ [50]–[51].

Of course, identifying rare variants requires genotyping large populations of individuals, which is costly and time-consuming, especially when one seeks to study large candidate gene sets typically involved in complex diseases. An interesting approach to minimize cost and time, is through sample pooling that in combination with massively parallel sequencing allows the identification and simultaneous quantification of (rare) variants in multiple individuals [52]–[53].

In this study, we have used multiplex PCR combined with next generation sequencing of pooled DNA, to explore the mutation burden in DISC1 and 10 of its interaction partners (ATF5, GRB2, FEZ1, LIS-1 (encoded by *PAFAH1B1*), PDE4B, NDE1, NDEL1, TRAF3IP1, YWHAE, and ZNF365) in schizophrenia patients versus control individuals. The candidate genes were first selected based on their convincingly reported interaction with DISC1. Next, both some more established/studied interactors – having (suggestive) evidence for involvement in psychiatric illness – and some new, potentially interesting candidates were included (Table S1) [36], [54].

## Results

### 454 sequencing validation and variant discovery

454 sequence analysis was performed on 4 DNA pools and on 1 individual patient DNA sample. The average number of mapped reads per amplicon was comparable between patient and control pools (1356 versus 1368 reads/amplicon, respectively (∼34 reads/individual)), justifying a comparison between the patient and control sequencing data.

The number of reads was homogeneously distributed over the different amplicon pools, except for multiplex reaction 12, where a lower number of reads was obtained (Figure S1 and Text S1, Results & Discussion).

To evaluate the performance of our pooling approach we compared the minor allele frequencies of the variants in the pooled samples (as determined by GS-FLX sequencing) with their actual frequencies for each of the validated variants. The observed and predicted frequencies correlated very well (R^2^ = 0.98) across a wide range of frequencies, demonstrating the accuracy of our DNA sample pooling (Figure S2). In addition, the incidence of false negatives was estimated by Sanger sequencing of a representative subset of amplicons (Table S3). Results indicated that the occurrence of type II errors was negligible as long as sequence coverage was sufficient (≥500 reads/amplicon). More details and additional performance measures of our experimental platform are provided in Supporting Text S1 (Results section).

Manual curation of the variants using NovoSNP 4.0, resulted in a total of 110 potential variants with a frequency ≥0.8% (in the discovery sample), of which 61 were located in the coding and untranslated regions of the target genes. These 61 variants were further validated by SNP genotyping in the complete association sample, (comprising 486 unrelated SZ patients and 514 unrelated control individuals) (57 variants), or Sanger sequencing in the original subject population (80 patient and 80 control samples) (4 variants), resulting in a final set of 50 confirmed coding and UTR variants (Table 1).

**Table 1:** Validated variants and their frequencies.

Chrom. location^a^	Alleles^b^	Type	Reference sequence^c^	Annotation^d^	dbSNP N°	Minor allele frequency			Genotype distribution		
						co (%)	SZ (%)	P	co	SZ	P
***ATF5***											
chr19∶55125979	G>A	Cs	ENSP00000307356	p.G20G		1.28	0.62	0.117	0/13/495	0/6/480	0.123
chr19∶55127810	C>T	Cs	ENSP00000307356	p.C166C	rs34877198	8.08	8.35	0.839	5/71/425	4/72/403	0.901
chr19∶55127811	C>T	CM	ENSP00000307356	p.R167C		0.20	0.51	0.281	0/2/503	0/5/481	0.228
chr19∶55127927	C>T	Cs	ENSP00000307356	p.T205T	rs61742136	1.79	1.15	0.212	0/18/484	0/11/469	0.229
chr19∶55128172	G>A	3′UTR	ENST00000306139	c.860G>A		0.88	0.21	0.069	0/9/504	0/2/483	**0.034**
chr19∶55128183	G>A	3′UTR	ENST00000306139	c.871G>A*	rs8667	33.66	35.8	0.318	53/236/219	74/200/212	**0.048**
***DISC1***											
chr1∶229829230	G>A	5′UTR	ENST00000295051	c.-7G>A	rs3738399	0.69	0.71	0.945	0/4/286	0/4/278	0.968
chr1∶229896375	C>T	CM	ENSP00000295051	p.A83V	rs76175896	1.37	2.06	0.299	0/14/496	1/18/466	0.374
chr1∶229896606	G>T	CM	ENSP00000295051	p.W160L		0.31	0.64	0.322	0/3/483	0/6/461	0.282
chr1∶229896918	G>A	CM	ENSP00000295051	p.R264Q*	rs3738401	30.16	30.38	0.925	44/213/242	41/209/229	0.954
chr1∶229969633	C>T	Cs	ENSP00000295051	p.L465L*	rs3738402	4.17	4.99	0.407	3/36/464	1/46/434	0.249
chr1∶229973212	C>T	Cs	ENSP00000295051	p.I469I	rs2492367	10.48	10.88	0.783	8/88/400	8/88/382	0.960
chr1∶229997671	T>C	splice	ENSG00000162946	g.169488T>C	rs2273890	13.82	11.91	0.227	9/118/365	14/83/369	**0.039**
chr1∶230020724	C>T	CM	ENSP00000295051	p.L607F*	rs6675281	18.90	17.23	0.360	19/148/325	17/129/327	0.592
chr1∶230020768	G>A	Cs	ENSP00000295051	p.L621L	rs12133766	6.92	5.19	0.108	2/66/438	4/42/436	0.065
chr1∶230069015	A>G	3′UTR	OTTHUMT00000092356	c.2105A>G*	rs3082	36.31	35.08	0.583	73/212/208	52/230/194	0.093
chr1∶230211221	A>T	CM	ENSP00000295051	p.S704C*	rs821616	29.10	29.34	0.907	35/221/244	46/182/239	0.115
chr1∶230211362	G>C	CM	ENSP00000295051	p.E751Q		0.80	1.45	0.210	1/6/494	0/14/469	0.071
chr1∶230239137	G>A	Cs	ENSP00000295051	p.E834E	rs41271517	0.00	0.10	0.489	0/0/492	0/1/476	0.492
***FEZ1***											
chr11∶124856682	A>T	CM	ENSP00000278919	p.D123E*	rs597570	17.43	18.71	0.472	16/142/341	20/140/321	0.693
chr11∶124827464	C>G	CM	ENSP00000278919	p.E358Q		1.77	1.13	0.206	1/16/492	0/11/474	0.439
***GRB2***											
no coding or UTR variants detected											
***NDE1***											
chr16∶15692550	C>T	CM	ENSP00000345892	p.T191I		1.41	1.26	0.754	1/12/485	1/10/466	0.948
chr16∶15698108	C>T	Cs	ENSP00000345892	p.Y279Y	rs17283846	6.00	3.70	**0.016**	1/57/434	0/35/438	**0.025**
chr16∶15725642	A>C	3′UTR	ENST00000342673	c.1041A>C	rs2075511	49.18	48.84	0.898	132/217/140	106/253/117	**0.022**
***NDEL1***											
chr17∶8311052	C>T	CM	ENSP00000333982	p.P342S		0.20	0.00	0.504	0/2/498	0/0/485	0.500
***PAFAH1B1***											
chr17∶2531863	C>T	3′UTR	ENST00000006951	c.1250C>T	rs6628	no frequency data					
***PDE4B***											
chr1∶66485784	C>G	CM	ENSP00000332116	p.A112G		0.60	1.04	0.269	0/6/498	0/10/469	0.258
chr1∶66603958	G>A	Cs	ENSP00000332116	p.E435E*	rs783036	42.68	43.56	0.695	92/224/162	92/220/151	0.942
chr1∶66607183	G>A	Cs	ENSP00000332116	p.R596R		0.79	0.73	0.850	0/8/496	0/7/475	0.862
chr1∶66610664	C>T	Cs	ENSP00000332116	p.L642L		1.29	1.56	0.704	0/13/490	0/15/467	0.618
***TRAF3IP1***											
chr2∶238894111	A>C	Cs	ENSP00000362424	p.T23T	rs13398676	24.28	25.86	0.448	23/172/254	41/129/238	**0.006**
chr2∶238902127	G>A	CM	ENSP00000362424	p.R139Q	rs61742338	3.16^e^	1.92^ f^	0.731	0/5/74	0/3/75	0.719
chr2∶238902490	A>G	CM	ENSP00000362424	p.N228S	rs3769110	4.19	4.04	0.858	2/38/461	3/33/447	0.802
chr2∶238902585	G>A	CM	ENSP00000362424	p.E260K		0.10	0.21	0.623	0/1/503	0/2/482	0.531
chr2∶238902692	G>T	CM	ENSP00000362424	p.K295N*	rs12464423	29.78	30.81	0.616	42/212/243	52/193/236	0.437
chr2∶238902713	T>C	Cs	ENSP00000362424	p.P302P	rs17854985	4.32	4.00	0.873	2/39/454	3/32/440	0.453
chr2∶238917916	A>C	CM	ENSP00000362424	p.D400A	rs61756349	0.00 e	0.63 e	0.316	0/0/80	0/1/79	0.317
chr2∶238917963	A>T	CM	ENSP00000362424	p.T416S	rs58277463	4.32	4.18	0.895	2/39/457	3/34/440	0.811
chr2∶238971007	A>C	CM	ENSP00000362424	p.M620L	rs3739070	13.82 e	10.26 e	0.432	3/15/58	0/16/62	0.208
chr2∶238972267	G>delG	CF	ENSP00000362424	p.V682Xfs		0.63 e	0.63 e	1.000	0/1/79	0/1/79	1.000
***YWHAE***											
chr17∶1212035	C>T	Cs	ENSP00000264335	p.K94K	rs34137556	4.00	2.95	0.210	2/36/462	0/28/447	0.316
chr17∶1250208	C>G	5′UTR	ENST00000264335	c.-54C>G		2.68	3.40	0.354	0/27/476	0/33/451	0.353
chr17∶1250195	G>A	5′UTR	ENST00000264335	c.-41G>A		1.28	1.24	0.920	0/13/496	0/12/472	0.940
***ZNF365***											
chr10∶64084647	C>T	CM	ENSP00000378672	p.P26L		0.10	0.32	0.332	0/1/496	0/3/472	0.285
chr10∶64085190	G>A	CM	ENSP00000378672	p.A62T	rs7076156	29.22	28.27	0.648	42/210/251	47/178/256	0.289
chr10∶63889533	C>T	Cs	ENSP00000342563	p.L318L		0.10	0.00	0.524	0/1/502	0/0/482	1.000
chr10∶63889538	G>A	Cs	ENSP00000342563	p.V319V		0.20	0.21	0.955	0/2/510	0/2/483	0.955
chr10∶63829339	T>G	CM	ENSP00000378674	p.S337A*	rs3758490	42.02	38.28	0.091	88/245/168	73/223/186	0.259
chr10∶64100025	C>T	Cs	ENSP00000342563	p.H449H		1.96	1.65	0.552	0/20/491	0/16/469	0.603
chr10∶63829578	T>C	3′UTR	ENST00000395254	c.1248T>C	rs41307502	0.99	1.03	0.921	0/10/497	0/10/475	0.920

Abbreviations: CM, missense; Cs, silent; CF, frameshift; co, control individuals; SZ, schizophrenia patients.

Significant values (P<0.05) are shown in bold. Variants and genotypes that are uniquely present in patients or control individuals are underlined.

*variants detected in the test DNA sample.

a Chromosomal positions from NCBI build 36.

b Major allele > minor allele on the + strand (http://www.genome.ucsc.edu).

c Reference sequences from Ensembl release 52 (December 2008).

d Annotations relative to the given reference sequence, with p. = protein reference sequence; c = coding DNA reference sequence; g. = genomic reference sequence. For coding DNA reference sequences, positions are relative to the ATG translation initiation codon.

e Frequencies determined by Sanger sequencing (80 samples).

### Variant frequency analyses

Variant frequencies of the 50 confirmed variants are listed in Table 1.

The genotype distribution of variant PAFAH1B1 r.1250C>T (rs6628) was not in HWE, and was omitted from further statistical analyses.

#### Main effect analysis

At the allelic level, only 1 variant, NDE1 p.Y279Y ( = rs17283846), showed a significant difference between patients and control individuals, exhibiting a higher frequency in the latter (Table 1; allelic OR = 0.60 (95% CI: 0.39–0.92), p = 0.016). At the genotypic level, 6 statistically significant effects were found, the strongest of which was observed for TRAF3IP1 p.T23T ( = rs13398676), showing a higher proportion of MAF homozygotes in patients versus controls (Table 1; OR = 2.07 (95% CI: 1.2–3.5), p = 0.008). However, none of these effects passed Bonferroni correction.

Interestingly, it was observed that large part of the identified coding variants represented rare mutations, with over 35% of the variants (18/50) having a MAF below 1%, and 50% (25/50) having a MAF smaller than 2%. Some of these variants were uniquely present in patients (DISC1 p.E834E,  = rs41271517) or in controls (NDEL1 p.P324S; ZNF365 p.L318L).

#### Mutation burden analysis

The overall variation burden (*i.e.* comprising all 49 variants in HWE) did not differ between the 486 patients and 514 control individuals genotyped, neither in the complete set of candidate genes (p = 0.75), nor on an individual gene basis (smallest p = 0.42). However, when the variants were stratified based on MAF and variant type, several interesting effects were observed (Table 2 and 3). It was found that the rare mutations (MAF <0.01) were more common in the patient population compared to the controls (1.24-fold increase, P = 0.246). Though not statistically significant for the assembled rare mutations (including missense, silent and UTR variants), this increased burden became significant when only the missense mutations were considered (1.85-fold increase at MAF<0.01, P = 0.018). This effect gradually diminished when variants with a higher MAF were included (1.28-fold increase at MAF<0.02, P = 0.112; 1.10-fold increase at MAF<0.05, P = 0.479). The observed result, however, did not remain statistically significant after Bonferroni correction (16 tests, corrected P = 0.29).

**Table 2:** Mutation burden of identified variants in SZ patients and controls, stratified according to type and minor allele frequency.

Variant type	Frequency^a^	n^b^	Av. # variant alleles/individual^c^		SZ/co ratio^d^	P^e^
			co	SZ		
All variants	All	49	9.12	9.07	0.99	0.748
	<1%	16	0.11	0.14	1.24	0.246
	<2%	24	0.35	0.35	1.00	0.985
	<5%	31	0.82	0.82	1.00	1.000
CM + CF	All	23	4.19	4.17	1.00	0.895
	<1%	9	0.05	0.09	1.85	**0.018**
	<2%	12	0.14	0.17	1.28	0.112
	<5%	15	0.31	0.34	1.10	0.478
Cs + splice	All	18	2.50	2.41	0.96	0.283
	<1%	4	0.02	0.02	0.96	0.926
	<2%	8	0.15	0.12	0.82	0.239
	<5%	11	0.39	0.35	0.92	0.423
UTR	All	8	2.94	2.98	1.01	0.492
	<1%	3	0.04	0.03	0.73	0.360
	<2%	4	0.07	0.06	0.82	0.440
	<5%	5	0.13	0.13	0.99	0.986

Abbreviations: CM, missense; Cs, silent; CF, frameshift; co, control individuals; SZ, schizophrenia patients.

Significant values (P<0.05) are shown in bold.

a Minor allele frequencies in the control population.

b Number of mutations identified in each variant subgroup. Only variants in HWE are listed.

c Mutation burden, defined as the average number of variant alleles/person.

d Fold increase of mutation burden in patients versus control individuals.

e Empirical P-values, obtained by performing 100000 permutations.

**Table 3:** Burden of rare missense variants in controls and SZ patients, stratified according to age at disease onset.

AAO	N^a^	Av. # variant alleles/individual^b^		SZ/co ratio^c^	P^d^
		co	SZ		
≤20	163	0.05	0.13	2.75	**0.0004**
21- <35	266	0.05	0.08	1.69	0.076
35- <60	57	0.05	0.00	0.00	0.15

Abbreviations: AAO, age at onset; CM, missense; CF, frameshift; co, control individuals; SZ, schizophrenia patients.

Significant values (P<0.05) are shown in bold.

a Number of individuals in each patient subgroup.

b Mutation burden, defined as the average number of variant alleles/person.

c Fold increase of mutation burden in patients versus control individuals.

d Empirical P-values, obtained by performing 100000 permutations.

Yet, upon further stratification of the data according to disease onset age, we found that the observed increase in rare missense mutation burden may not be a general effect in our SZ sample, but seems to be related to the age at disease onset, being most pronounced in patients with a young onset age.

More specifically, the patient population was stratified into three groups: early-onset (≤20) (N = 163), medium-onset (21 - <35) (N = 266) and late-onset (35 - <60) (N = 57) patients, and the burden of rare missense mutations (MAF <1%) was investigated in these groups. It was found that the early-onset patients had a particularly high burden of these rare missense variants: in this subset of patients, we found 12.9 mutant alleles per 100 individuals, versus 8.6 in the complete SZ sample and 4.7 in controls (SZ/co ratio  = 2.75; P = 0.0004). This burden decreased to 7.9 mutant alleles per 100 individuals in the medium onset group (SZ/co ratio  = 1.69; P = 0.076), and was zero in the late-onset group (0 mutant alleles/100 individuals; P = 0.15).

The observed increased burden of rare missense mutations in early onset patients also remains significant after Bonferroni correction (19 tests, corrected P = 0.0076).

### *In silico* functional analyses

For all variants, the degree of nucleotide conservation was assessed using the GERP (Genome Evolutionary Rate Profiling) score. Missense and silent mutations were further examined for potential splicing defects. Several potential effects on splicing were predicted, with varying consistencies across the different matrices (Table 4 and 5). These include the disruption of several potential ESE sites, and the creation of two potential new splicing motifs in *DISC1* (one donor and one acceptor site), which were predicted by both of the splice-site prediction algorithms used. Neither of these two alternative splice variants corresponded to the splice isoforms described by Nakata and co-workers[55]. . The strongest evidence – i.e., corresponding to the highest number of predictions, across multiple matrices – was found for TRAF3IP K295N ( = rs12464423) (a common variant; OR = 1.05, 95% CI: 0.87–1.28), and FEZ1 E358Q, a rare variant which is slightly more present in control individuals compared to patients (OR = 0.64, 95% CI:0.30–1.40).

**Table 4:** Properties and potential functional effects of missense and frameshift mutations.

Annotation^a^	Novel?	OR (95% CI)^c^	NT conservation^d^	Splicing analysis^e^	AA conservation^f^			Interpretation^g^
			GERP	Predicted effect(# predictions/# matrices)	SIFT	Polyphen	Panther	
***ATF5***								
R167C	Y	**2.61 (0.5–13.4)**	0		++	(++)	+	**Possible amino acid effect**
***DISC1***								
A83V		**1.51 (0.75–3.01)**	**+**	new splice site [−801 nt] (2/2)	+	−	−	**Possible splicing effect**
W160L	Y	**2.09 (0.52**–**8.37)**	0		−	−	−	No obvious functional effect
R264Q		1.01 (0.83–1.22)	−	new splice site [−725 nt] (2/2)	−	−	−	**Possible splicing effect**
L607F		0.89 (0.7–1.12)	**+**		+	−	+	**Possible amino acid effect**
S704C		1.01 (0.83–1.23)	**+**		+	+	+	**Possible amino acid effect**
E751Q b		1.83 (0.76–4.37)	**+**		+	−	−	No obvious functional effect (though genomically conserved)
***FEZ1***								
D123E		1.09 (0.86–1.37)	0		−	−	−	No obvious functional effect(though genomically conserved)
E358Q	Y	**0.64 (0.29–1.35)**	**+**	ESE site broken (8/4)	−	−	−	**Possible splicing effect**
***NDE1***								
T191I	Y	0.89 (0.41–1.94)	**+**		−	−	−	No obvious functional effect (though genomically conserved)
***NDEL1***								
P342S	Y	**0.00 (0.00-NaN)**	**+**		(+)	(++)	NM	Uninterpretable*
***PDE4B***								
A112G	Y	**1.76 (0.63–4.86)**	**+**		−	−	NM	No obvious functional effect (though genomically conserved)
***TRAF3IP1***								
R139Q	Y	0.60 (0.14–2.55)	0		−	−	NM	No obvious functional effect
N228S		0.96 (0.61–1.5)	−		−	−	NM	No obvious functional effect
E260K	Y	**2.08 (0.19–23.03)**	**+**		−	−	NM	No obvious functional effect (though genomically conserved)
K295N		1.05 (0.86–1.27)	0	ESE site broken (13/4)	(+)	−	NM	**Possible splicing effect** *
D400A		**Inf (NaN-Inf)**	**+**		(−)	(++)	NM	No obvious functional effect (though genomically conserved)*
T416S	Y	0.96 (0.62–1.5)	0		(−)	−	NM	Uninterpretable*
M620L		0.71 (0.36–1.43)	**+**		−	−	NM	No obvious functional effect (though genomically conserved)
V682X	Y	1.00 (0.06–16.13)	**+**		NA	NA	NA	**Possible functional effect (premature stop codon)**
***ZNF365***								
P26L	Y	**3.15 (0.32–30.2)**	0		(+)	−	−	No obvious functional effect
A62T		0.95 (0.78–1.16)	−		(−)	−	−	No obvious functional effect
S337A		0.86 (0.71–1.02)	0		−	−	−	No obvious functional effect

a Rare mutations (MAF<1%) are underlined.

b Rare allele, also reported by Song and colleagues^74^.

c OR>1.5 or <0.67 are shown in bold.

d GERP: -, divergent (score <−1); +, conserved (score >1); 0, intermediate (−1< score <1).

e Only splicing predictions based on >1 matrix, and located <30 nt from the nearest exon-intron boundary were considered. For predicted splice sites, exon length variation associated with the use of the cryptic site is indicated between square brackets.

f SIFT: -, tolerated; + possibly damaging; ++, damaging. PolyPhen: -, benign; +, possibly damaging; ++, probably damaging. Panther: -, unlikely functional effect (pdeleterious <0.5); +, possibly damaging (0.5 < pdeleterious <0.75); NM, not modeled by the algorithm.

AA conservation predictions in parenthesis are based on less than 6 sequences in the alignment, and should be interpreted with caution.

NA: not applicable.

g Interpretations were made irrespective of the observed odds ratios.

*more sequence data needed to allow more reliable predictions to be made on the amino acid level.

**Table 5:** Properties and potential functional effects of silent and splice site mutations.

Annotation^a^	Novel?	OR (95% CI)^b^	NT conservation^c^	Splicing analysis^d^	Interpretation^e^
			GERP	Predicted effect(# predictions/# matrices)	
***ATF5***					
G20G	Y	**0.48 (0.18–1.27)**	0		No obvious functional effect
C166C		1.04 (0.75–1.43)	0		No obvious functional effect
T205T		**0.63 (0.29–1.35)**	0		No obvious functional effect
***Disc1***					
I469I		1.04 (0.78–1.39)	−	ESE site broken (2/2)	**Possible splicing effect**
L465L		1.21 (0.78–1.84)	−		No obvious functional effect
L621L		0.74 (0.5–1.07)	+		No obvious functional effect (though genomically conserved)
g.169488T>C		0.84 (0.64–1.1)	+		**Possible splicing effect (5**′ **splice site)**
E834E		**Inf (NaN-Inf)**	**+**		No obvious functional effect (though genomically conserved)
***NDE1***					
p.Y279Y		**0.60 (0.39–0.92)**	−		No obvious functional effect
***PDE4B***					
E435E		1.04 (0.86–1.24)	−	ESE site broken (2/2)	**Possible splicing effect**
R596R	Y	0.91 (0.33–2.53)	−		No obvious functional effect
L642L	Y	1.21 (0.57–2.55)	0		No obvious functional effect
***TRAF3IP1***					
T23T		1.09 (0.87–1.35)	−		No obvious functional effect
P302P		0.96 (0.61–1.51)	−	ESE site broken (4/2)	**Possible splicing effect**
***YWHAE***					
K94K		0.73 (0.44–1.19)	+		No obvious functional effect (though genomically conserved)
***ZNF365***					
L318L	Y	**0.00 (0.00-NaN)**	**+**		No obvious functional effect (though genomically conserved)
V319V	Y	1.06 (0.14–7.51)	−		No obvious functional effect
H449H	Y	0.84 (0.43–1.63)	−		No obvious functional effect

a Rare mutations (MAF<1%) are underlined.

b OR>1.5 or <0.67 are shown in bold.

c GERP: -, divergent (score <−1); +, conserved (score >1); 0, intermediate (−1< score <1).

d Only splicing predictions based on >1 matrix, and located <30 nt from the nearest exon-intron boundary were considered. For predicted splice sites, exon length variation associated with the use of the cryptic site is indicated between square brackets.

e Interpretations were made irrespective of the observed odds ratios.

The variants causing amino acid substitutions were further evaluated by analyzing evolutionary amino acid conservation using SIFT, PolyPhen and Panther. The resulting conservation scores indicated that the majority of identified amino acid substitutions in our data set had either little or no functional effect (scored as neutral by at least two these algorithms), or required more homologous sequence data in order to make reliable predictions. Two variants however, were concordantly predicted to be potentially damaging: ATF5 p.R167C (SIFT, damaging; PolyPhen, probably damaging; Panther, possibly damaging) and DISC1 p.S704C ( = rs821616) (all three algorithms: possibly damaging). In addition, one variant (DISC1 p.L607F) ( = rs6675281) was predicted to be possibly damaging by SIFT and Panther, but scored as benign by PolyPhen (Table 4).

The missense mutations were also examined for putative disruptions of specific structural and functional properties using various predictors in the SNPeffect toolsuite (Reumers et al., 2008). Single amino acid replacements can affect the protein's structure and dynamics, they can disrupt functional sites or affect the cellular processing of the protein. None of the missense variants detected in this study caused significant changes in any of the properties examined. The lack of three dimensional structures of the proteins in the DISC1 pathway, or even structural models of close homologs, hindered a detailed analysis of the influence of the mutations on protein stability.

**Figure 1:**
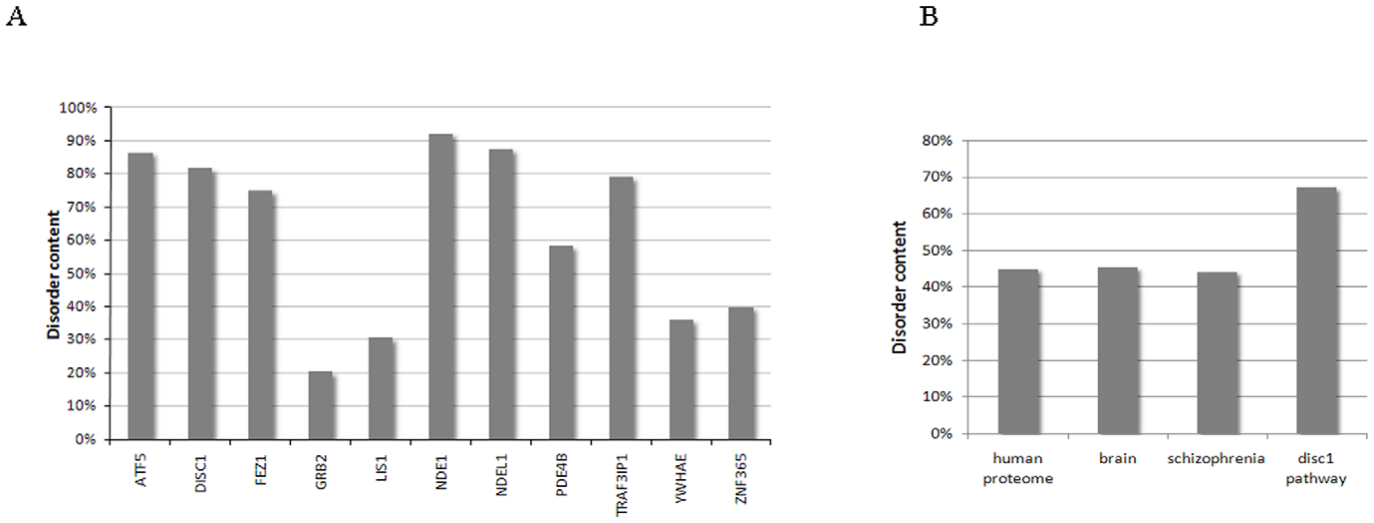
Prevalence of intrinsic disorder in the studied DISC1 pathway proteins, as measured by DisProt analysis. (A) Disorder content of the individual proteins of the DISC1 pathway (i.e. percentage of disordered residues/ total number of amino acids). (B) Disorder content of the DISC1 pathway proteins compared to human proteome (i.e. 20320 SwissProt proteins), a set of brain proteins (7160 sequences from GeneAtlas) and a set of schizophrenia candidate proteins (670 sequences from the Schizophrenia Gene Resource database). Overall, the DISC1 pathway proteins exhibit a higher abundance of intrinsically disordered residues, compared to the human proteome.

However, this scarcity of structural models is probably due to a high occurrence of intrinsically disordered regions in the proteins under study. Indeed, DisProt analysis showed that all proteins except LIS1 (encoded by *PAFAH1B1*), GRB2 and YWHAE have ≥40% disordered residues (Figure 1) (Peng et al., 2006). The missense variants were overrepresented in proteins with a high content of unstructured regions, with 19 of the 22 identified missense mutations (86%) residing in an unstructured region of the protein, and only 3 mutations (DISC1 L607F ( = rs6675281), DISC1 S704C ( = rs821616) and ZNF365 A26T ( = rs7076156)) lying outside such a region (Figure S4). Notably, none of the 3 more ‘structured’ proteins contained missense mutations.

Finally, all UTR variants were analyzed for interference with predicted transcription factor binding sites, miRNA target sites. We did not find evidence for mutations affecting transcription factor binding sites, but we did identify three potential miRNA target site mutations, in the 3′ UTR of *ATF5* and *DISC1* (Table 6).

**Table 6:** Properties and potential functional effects of UTR mutations.

Annotation^a^	Novel?	OR (95% CI)^b^	NT conservation^c^	Predicted motif	Predicted target location	Score	P	Interpretation
***ATF5***								
r.860G>A	Y	**0.23** (0.05–1.08)	0	hsa-miR-193b target^d^	chr:1955128171–55128193	15.71	0.002	**Potential miR target disrupted**
r.871G>A		1.10 (0.91–1.32)	−	hsa-miR-193b target^d^	chr:1955128171–55128193	15.71	0.002	**Potential miR target disrupted**
***Disc1***								
r.-7G>A		1.03 (0.26–4.13)	0					No obvious functional effect
r.2105A>G		0.95 (0.79–1.14)	**+**	hsa-miR-633 target	chr:1230069003–230069025	16.40	0.035	**Potential miR target disrupted**
				hsa-miR-30d* target^d^	chr:1230069012–230069032	16.60	0.047	
***NDE1***								
r.1041A>C		0.99 (0.82–1.17)	−					No obvious functional effect
***PAFAH1B1***								
r.1250C>T			+					No obvious functional effect (though genomically conserved)
***YWHAE***								
r.-41G>A	Y	0.97 (0.44–2.14)	**+**					No obvious functional effect (though genomically conserved)
r.-54C>G	Y	1.28 (0.76–2.14)	+					No obvious functional effect (though genomically conserved)
***ZNF365***								
r.1248T>C		1.05 (0.43–2.52)	−					No obvious functional effect

a Rare mutations (MAF<1%) are underlined.

b OR>1.5 or <0.67 are shown in bold.

c NT conservation assessed by the GERP score: -, divergent (score <−1); +, conserved (score >1); 0, intermediate (−1< score <1).

## Discussion

To explore the role of genetic variation in *DISC1* and 10 of its interaction partners in the etiology of schizophrenia, we sequenced the coding exons and splice junctions of the genes using massively parallel 454 sequencing in pooled samples. A selection of 80 early onset SZ patients and 80 control individuals was used as a discovery sample, resulting in the identification of 50 validated variants (Table 1). These 50 variants were subsequently genotyped in the complete association samples comprising 486 SZ patients and 514 control individuals recruited from an isolated northern Swedish population.

Six variants were found with a statistically significant frequency difference between patients and controls. These include two synonymous mutations, NDE1 Y279Y ( = rs17283846) (p = 0.025) and TRAF3IP1 T23T ( = rs13398676) (p = 0.006). Although the functional consequences of silent mutations may not be obvious, recent studies have shown that these variants might modify protein abundance, structure and/or activity via alterations in mRNA stability [56], splicing [57], or translation kinetics[58]. The other variants showing a significant effect in two or more of the inheritance models all involve UTR or splice site mutations: *ATF5* r.871G>A, *DISC1* g.169488T>C ( = rs2273890) and *NDE1* r.1041A>C ( = rs2075511). Interestingly, *ATF5* r.871G>A is located in a potential miRNA target site, and may therefore interfere with ATF5 expression and function. However, as none of these effects survived multiple testing correction, further independent replication of these findings will be required.

The observed scarcity of statistically significant main effects in our data set does not necessarily rule out the involvement of the DISC1 pathway in the susceptibility for SZ in our population, but may (at least partly) be attributed to the relatively high occurrence of rare variants, having frequencies too low to be able to run adequate statistical comparisons. Indeed, over 35% of all identified coding variants (18/50) have a MAF below 1%, and 50% (25/50) were present at a MAF smaller than 2%. Though the frequencies of these rare variants were not significantly different between patients and controls, they often have odds ratios higher than 1.5 (resp. lower than 0.67), and several are unique in one or the other group (Tables 4, 5, 6). As for *DISC1*, none of rare variants identified here overlapped with the 5 ultra-rare cohort-specific variants previously described by Song et al. [59]. Of the two rare variants we identified in this gene, one (W160L) was completely novel, and the other (E751Q) was also reported as rare by Song and colleagues (MAF<1%). Interestingly, this variant also had an OR of ∼2 in their population, analogous with our results (Table 4).

To understand the potential role of the identified rare variants in SZ etiology, we evaluated the mutation burden (defined as the average number of mutations per person) in patients versus controls. Under a model in which rare mutations increase risk, we would expect to observe a greater burden in patients compared to control individuals. There was no difference in overall mutation burden between the genotyped patients and control individuals. Yet, when mutation burden was defined not simply as the total number of variants – including neutral polymorphisms – but evaluated as subgroups of variants (based on MAF and variant type), we found that schizophrenia patients were 1.85 times as likely as controls to harbor rare variants (MAF<0.01) causing amino acid substitutions (including frameshifts) (empirical P = 0.018, Table 2), indicating a role for these variants in SZ etiology in at least the northern Swedish population. Though this effect was no longer significant after stringent Bonferroni correction, it pointed us towards an even stronger effect in a subgroup of patients. Indeed, the observed increased burden of rare missense mutations seems to be related to the age at disease onset, being most pronounced in patients with a young onset age, having a 2.75-fold higher burden of rare missens variants compared to controls (empirical P = 0.0004, Bonferroni corrected P = 0.0076) (Table 3). This observation is in line with previous clinical, cognitive, genetic and imaging studies, implicating that early onset SZ is associated with greater genetic loading [60]–[65], overabundance of rare CNVs impacting on known genes [66] and increased neurodevelopmental deviance [65], amongst others. These data emphasize the importance of studying subgroups of patients and identifying endophenotypes.

In addition, our findings support the hypothesis that multiple, individually rare mutations contribute to SZ risk [50] and, given the distribution of the variants across different genes, also explain the allelic and locus heterogeneity typically observed in SZ. Replication of our findings in larger sample sets will however be required to further substantiate the observed effects.

Detailed analysis of the variants contributing to the increased burden, showed that 8 out of 9 identified rare non-synonymous mutations in this study had an increased abundance in patients versus controls. These 8 mutations are located in *DISC1* (2 mutations), *PDE4B* (1 mutation), *ATF5* (1 mutation), *TRAF3IP1* (3 mutations) and *ZNF365* (1 mutation) (Table S4). Though not statistically significant on a single gene level, each of these genes causes an individual increase in mutation burden with a factor ∼2 in patients versus controls (average fold increase 2.24±0.51). Taking into account the number of coding bases in these genes, *DISC1* and *ATF5* were found to have highest mutation burden per base (Table S4), and may thus be considered the strongest candidates for further detailed mutation analysis in a larger sample. Indeed, only a subset of our patient population (80 individuals out of 486) was sequenced in this study, enabling the detection of merely a fraction of all rare variants present in this population (see Supporting Text S1, Discussion section). Follow-up sequencing of the candidate genes in the complete patient sample may therefore uncover other rare (non-synonymous) mutations, possibly further contributing to the observed differences in mutation burden.

In order to estimate the potential risk associated with the identified missense variants, a range of protein structural and functional properties was investigated. Rather unexpectedly, we found that none of the 22 identified missense variants caused any significant effect on the various properties examined. This absence led us to the observation that 8 of 11 proteins under study showed a remarkably high occurrence of intrinsically disordered regions (IDRs). Indeed, all proteins except LIS1, GRB2 and YWHAE were found to have ≥40% of disordered residues by DisProt analysis (Figure 1, panel A). Furthermore, we observed that ∼90% of the identified missense variants were located in these IDRs (Figure S4), while neither *PAFAH1B1*, *GRB2,* nor *YWHAE* contained a single missense variant.

IDRs are segments of proteins that do not definitively fold and remain flexible and unordered. These proteins take up different structures upon binding to different targets, and thereby exhibit functional flexibility [67]–[68]. Disordered regions of proteins have been shown to have important physiological roles, including molecular recognition, cell regulation and signal transduction [69]. It is therefore not surprising that protein disorder turns out to be very common in human diseases – being significantly enriched in a wide variety of disease-associated proteins, including neurodegenerative disease, cancer, cardiovascular disease and diabetes [70]–[75]. Furthermore, it has been shown that IDRs are particularly prevalent in hub proteins and interaction networks, where their conformational flexibility is required to accommodate binding between the different interaction partners [76]–[78]. Interestingly, our analyses revealed that this is also the case here, with the DISC1 pathway proteins clearly exhibiting a higher abundance of intrinsically disordered residues, compared to the human proteome , as well as a set of brain and schizophrenia-related protein sequences (p = 0.018; 0.013 and 0.0098, respectively) (Figure 1, panel B). This is an exciting new insight, which – to our knowledge – has never been reported in the literature, and may provide a new boost to the complex research field of psychiatric genetics. While alterations of disordered regions may not directly cause changes in protein structure, they are very well capable of interfering with the function of proteins[79], e.g. by affecting the affinity for interaction with other proteins, or altering the coupled binding-folding mechanism of (one of) the binding partners. Importantly, it has been shown that intrinsically disorder is very sensitive to changes in amino acid sequence; as recently described [80]–[81], maintaining disordered regions through evolution (or sequence changes) appears very difficult, whereas helices and strands are maintained more easily. Neutral mutations with respect to disorder are therefore very unlikely [80]–[81]. Certainly in a complex network, such as the DISC1 pathway, it is very well conceivable that mutations and/or changes in one of the proteins or its environment could reduce its ability to recognize appropriate binding partners and lead to partial or complete collapse of the protein network.

In this study, ∼90% of all identified missense variants (including the rare mutations underlying the increased burden in patients versus controls) are located in an IDR. Interestingly, some of the (rare) variants indentified in this study fall into known binding regions on one or more of the interactors (Figure S3). E.g., ATF5 R167C is located in the DISC1 binding region of this protein; DISC E751Q resides in the binding sites for ATF5, LIS1 and PDE4B; and TRAF3IP1 E260K is located in the DISC1 binding region of this protein. Although these observations are certainly very intriguing, they should be regarded with some caution, as the reported binding regions between the different interactors are often quite large, hence no clear conclusions can be drawn from them. Moreover, as not all of the binding regions for the different interactions have been described in literature, it is impossible to give a complete picture of this. The question whether one (or more) of these mutations might influence protein (or even pathway) function, by interfering with any of the key features associated with IDRs, will be one of the major challenges for future work. A first clue about potential effects of some of the variants may be provided by their amino acid conservation (Tables 4, 5, 6). Based on evolutionary conservation scores generated by 3 different algorithms, we found that three variants were predicted to be possibly damaging: *ATF5* R167C, *DISC1* L607F ( = rs6675281) and S704C ( = rs821616). Interestingly, two of these variants (*DISC1* S704C and L607F) were recently reported to have an actual functional effect [82]–[84]. The fact that the predicted outcome for *DISC1* L607F and S704C corresponds to already known biological consequences greatly underlines the value of our *in silico* predictions, also for other, unknown variants. This is especially interesting as to *ATF5* R167C, which was also predicted to be damaging, but not previously reported. This variant corresponds to a novel, rare mutation, having an odds ratio of 2.6 (95% CI: 0.50–13.46). Further studies of this variant are warranted to clarify its relation to disease.

To our knowledge, this study is the first describing a comprehensive resequencing analysis of the DISC1 pathway in schizophrenia. Our results provide support for a model of SZ pathogenesis that includes the effects of multiple rare variants, residing in different vulnerable genes, which may in turn be functionally linked into pathways and networks. This model is consistent with the theory presented by Eyre-Walker [85], stating that rare alleles should explain most of the variance in complex traits if there is natural selection for the trait. Based on these findings, and as also suggested by McClellan and co-workers [50], we argue that rare risk alleles may be revealed by research strategies including extensive resequencing of genes previously shown to be informative (e.g. based on a chromosomal translocation, such as *DISC1*) and, importantly, these genes' functional network.

Assigning potential functional significance to identified variants is a major challenge in genomics research. In this work, a wide array of functional properties was examined to predict possible deleterious effects of the variants. Using these tools, we were able to predict several potential effects on splicing and miRNA target motifs. Yet, alterations of protein structure or function were hard to track down using standard *in silico* prediction programs, as a majority of the proteins encoded by our candidate genes contain large regions of intrinsically disordered residues. Though amino acid conservation analysis may provide a first hint of potential functional effects, it does not tell the whole story, as disorder-based signaling is a complex process, depending on multiple factors including alterations in protein context, alternative splicing and post-translational modifications [77], [86]. However, in our opinion, this high prevalence of IDRs in the DISC1 pathway is a very fascinating finding *in se*, hopefully encouraging further research into this complex area, and providing new clues to our understanding of the complex etiology of SZ and other (psychiatric) disorders. Indeed, as an increasing amount of evidence is beginning to emerge that many important biological functions depend directly on the disordered state, alteration of this disorder may play a crucial role in the pathogenicity of many complex diseases (including SZ), thereby adding another level of complexity to the study of their molecular mechanism, and providing exciting new perspectives for future research.

## Materials and Methods

### Subjects

In a first phase of the study we used DNA of 80 SZ patients (41 females, 39 males) and 80 control individuals (40 females, 40 males) for 454 sequencing based variant discovery. These individuals were selected on the basis of their early age at disease-onset (18.55±3.36), from a larger association sample consisting of 486 unrelated SZ patients (180 females, 306 males) and 514 unrelated control individuals (275 females, 239 males). All originated from a geographically isolated population living in the county of Västerbotten in Northern Sweden. They were all Caucasians and none were of Finnish, Norwegian or Lappish descent. All patients fulfilled the *DSM-IV* criteria for SZ [87]. The mean age at disease-onset in the complete SZ sample was 24.8 (±7.3) years and the mean age at inclusion 53.1 (±15.1) years (see Supporting Text S1 (Materials and Methods section) for additional information regarding the ascertainment and assessment procedure of the patients).

The control population had a mean age of 58.0 (±13.0) years at inclusion. They originated from the same geographical area as the patients and were randomly selected from the Betula study, described in detail elsewhere (http://www.betula.su.se/en/) [88]–[89]. None of the controls were reported to have a diagnosis of schizophrenia based on studies of psychiatric records and/or an interview.

All participants gave written informed consent, and the study was approved by the regional Medical Ethical Committees of the universities of Umeå and Antwerp.

The patient-control sample was controlled for population stratification by the genotyping of 37 microsatellite (STR) markers via the use of standard genotyping and scoring methods. Statistical tests for population stratification were performed using the program STRUCTURE (http://pritch.bsd.uchicago.edu/structure.html). No population substructure was observed in the association sample (data not shown).

### DNA samples and pooling

Genomic DNA was extracted from peripheral blood using standard methods.

4 DNA pools were prepared (2 ‘patient pools’ and 2 ‘control pools’), each comprising 40 DNA samples. Hereto, an equal amount of each sample (100 ng per individual) was combined, and the resulting DNA pool was adjusted to a final concentration of 10 ng/µL.

To control the efficiency of DNA pooling, the relative abundance of 3 SNP alleles was measured by pyrosequencing and compared to the allele frequencies of the individual samples constituting the pools (Supporting Text S1 (Materials and Methods section) and Table S2).

### Multiplex PCR reactions and 454 sequencing

Multiplex PCR assays were designed to amplify all coding exons and splice junctions of the 11 selected genes (totaling ∼16 kb target sequence). The target sequence was covered by 155 amplicons with an average length of ∼221 bp, resulting in ∼34 kb of sequence. The 155 amplicons were amplified in 12 multiplex PCR reactions. Simplex PCR reactions of the amplicons showed that all except two of the primer pairs (both in *ATF5*) amplified the correct fragment (conversion rate  = 98.7%). The two failed *ATF5* amplicons were omitted from further experiments. Multiplex PCR reactions were performed for each DNA pool and 1 individual patient DNA sample, also contained in one of the patient pools (Supporting Text S1 (Materials and Methods section).

Each multiplex PCR reaction was purified on a QIAquick PCR Purification column (Qiagen GmbH, Hilden, Germany), and the concentration of the eluates measured using a Nanodrop spectrophotometer (NanoDrop Technologies, Wilmington, DE). Finally, for each DNA pool (and the individual sample), the 12 purified multiplex PCR products were mixed, taking into account the concentration of each multiplex reaction and its number of constituent amplicons, to obtain an equal representation of every amplicon in the final PCR mixtures.

The final mix of 155 amplicons of each sample was sequenced using the standard amplicon sequencing protocol on a 454 GS-FLX genome sequencer (Roche Applied Science) according to the manufacturer's instructions. For each of the 4 pools, 1 lane of a 2-lane Bead Loading gasket on a 70×75 mm PicoTiterPlate was loaded, and sequenced from both directions. The individual DNA sample was sequenced using 1 lane of a 16-lane Bead Loading gasket.

### Variant detection and validation

The generated standard 454 flow files were analyzed using NovoSNP 4.0 (beta), an in-house developed software program for the identification of variants in resequencing experiments. In short, NovoSNP 4.0 uses the quality and height of the flow at the variation position and the neighboring flows for SNP identification. Further, it takes into account the number and ratio of reads showing the variant – thereby allowing for the analysis of pooled sequencing data – and favors variants seen in both directions. Finally, the program creates a database of all identified variants, for which the flows can be visually inspected (De Rijk P., personal communication).

All variants with a frequency ≥0.8% were examined, allowing for a secure cutoff level to detect singleton variants, which theoretically have a frequency of 1.25% in a pool of 40 samples.

Finally, all potential variants (except 4) were validated using iPLEX SNP genotyping in the complete association sample (486 patients and 514 control individuals). For technical reasons, 4 variants were genotyped by traditional Sanger-based sequencing in the original subject population (80 patient and 80 control samples) (Supporting Text S1 (Materials and Methods)).

### Statistical analyses

gPlink version 2.050 [90] (http://pngu.mgh.harvard.edu/purcell/plink/) was used to calculate genotype deviation from Hardy-Weinberg equilibrium (HWE), by an exact test [91], and to compare individual allele and genotype frequencies between patients and controls, by a standard χ2 test for independence.

Differences in mutation burden (defined as the average number of variant alleles per individual) between patients and controls were assessed by two-sided t-tests, using SPSS version 16.0.2 (Brussels, Belgium). The data were thereby stratified according to type (missense, silent and UTR variants) and MAF (<0.01, <0.02 and <0.05, respectively). Empirical p-values were generated using the max(T) permutation approach, based on 100000 replicates. The level of significance for all statistical tests was 0.05. When correcting for multiple testing, Bonferroni corrective measures were taken to control false positive rates. All association analyses were performed on the complete sample (i.e. including the discovery samples). Contrary to the use of patient samples *only* for variant discovery, inclusion of an equally large control sample in the discovery phase, does not lead to an inflation of type I errors [92] (see Supporting Text S1, Discussion section).

### *In silico* functional analyses

To investigate potential deleterious effects associated with the identified variants, each variant was subjected to a battery of *in silico* analyses, including assessment of nucleotide and amino acid conservation, effects on potential splice sites and *cis*-acting elements, potential disruption of miRNA target sites and predicted transcription factor binding sites, and alterations of functional and structural properties of the proteins. Finally, sequences where also examined for intrinsically disordered regions using DisProt (http://www.ist.temple.edu/disprot/Predictors.html). A detailed description of the applied methods is provided in Supporting Text S1.

## Supporting Information

Figure S1 **Boxplot showing the distribution of the number of reads per amplicon in each multiplex PCR reaction, for control and patient pools.** The observed read count is uniformly distributed across the different amplicon pools (with an average of 1362 reads/amplicon), except for multiplex reaction 12. °: outliers (values between 1.5 and 3x the interquartile range from either end of the box) *: extreme outliers (values more than 3x the interquartile range from either end of the box).
(PDF)

Figure S2 **Allele frequencies estimated from pooled DNA samples (as determined by GS-FLX sequencing) versus the actual frequencies (as determined by genotyping the individual samples) in the different pools.** °: outlier (corresponding to rs13398676, in SZ pool 1).
(PDF)

Figure S3 **Overview of the known interaction domains between the different proteins investigated, along with the positions of the variants identified in this study.** Protein lengths are given between brackets. Binding sites between two proteins are indicated along the line connecting them, with the binding site(s) on a certain protein closest to that protein (orange: binding sites on DISC1, blue: binding site on other proteins). The identified missense mutations are shown in a white area within the proteins' oval. Rare missense mutations (MAF ≤1%) are underlined, and mutations located in one of the binding sites are shown in italic. Note that the positions of many of the binding sites were not described/found in literature (indicated with ‘?’).
(PDF)

Figure S4 **Schematic representation of the overall domain architecture of each of the proteins investigated, highlighting the regions of predicted disorder (orange), along with regions having known homologous domains (blue).** On each protein, the identified missense mutations are indicated. Mutations lying outside a disordered region are marked with an extra line.
(PDF)

Table S1 **DISC1 interaction partners included in the study, and evidence for their involvement in psychiatric disease.**
(PDF)

Table S2 **Allele frequencies of pooled DNA and individual samples, as determined by pyrosequencing.**
(PDF)

Table S3 **Properties of amplicon subset selected for false negative rate estimation using Sanger sequencing.**
(PDF)

Table S4 **Mutation burden of identified rare nonsynonymous mutations (MAF<1%), stratified by gene.**
(PDF)

Text S1 **Supplementary Materials and Methods, Results and Discussion.**
(PDF)
