# Supplementary material for: Sequencing of DISC1 Pathway Genes Reveals Increased Burden of Rare Missense Variants in Schizophrenia Patients from a Northern Swedish Population
Source: PLoS One. 2011 Aug 11;6(8):e23450. doi: 10.1371/journal.pone.0023450 (PMC3154939; doi:10.1371/journal.pone.0023450)

Figure S1: Boxplot showing the distribution of the number of reads per amplicon in each multiplex PCR reaction, for control and patient pools. The observed read count is uniformly distributed across the different amplicon pools (with an average of 1362 reads/amplicon), except for multiplex reaction 12. °: outliers (values between 1.5 and 3x the interquartile range from either end of the box) *: extreme outliers (values more than 3x the interquartile range from either end of the box)

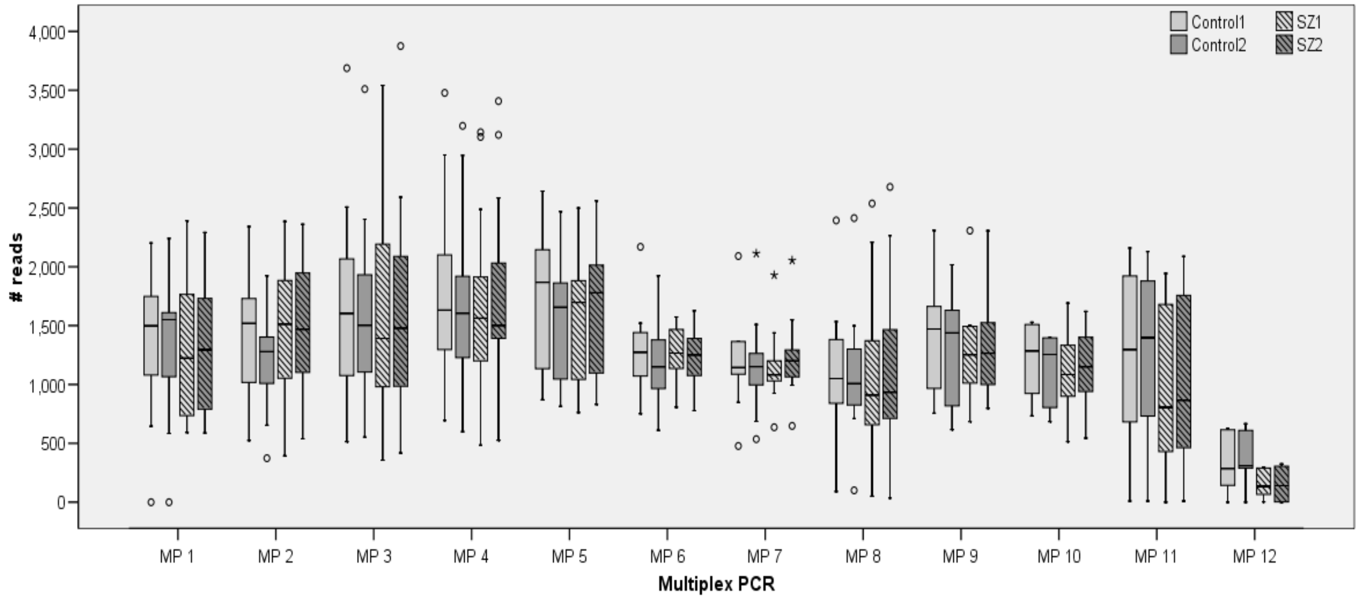

Supplement: Figure S1 — Boxplot showing the distribution of the number of reads per amplicon in each multiplex PCR reaction, for control and patient pools. The observed read count is uniformly distributed across the different amplicon pools (with an average of 1362 reads/amplicon), except for multiplex reaction 12. °: outliers (values between 1.5 and 3x the interquartile range from either end of the box) *: extreme outliers (values more than 3x the interquartile range from either end of the box). (PDF) [file pone.0023450.s001.pdf]
