# Supplementary material for: Sequencing of DISC1 Pathway Genes Reveals Increased Burden of Rare Missense Variants in Schizophrenia Patients from a Northern Swedish Population
Source: PLoS One. 2011 Aug 11;6(8):e23450. doi: 10.1371/journal.pone.0023450 (PMC3154939; doi:10.1371/journal.pone.0023450)

Figure S2: Allele frequencies estimated from pooled DNA samples (as determined by GS-FLX sequencing) versus the actual frequencies (as determined by genotyping the individual samples) in the different pools. °: outlier (corresponding to rs13398676, in SZ pool 1)

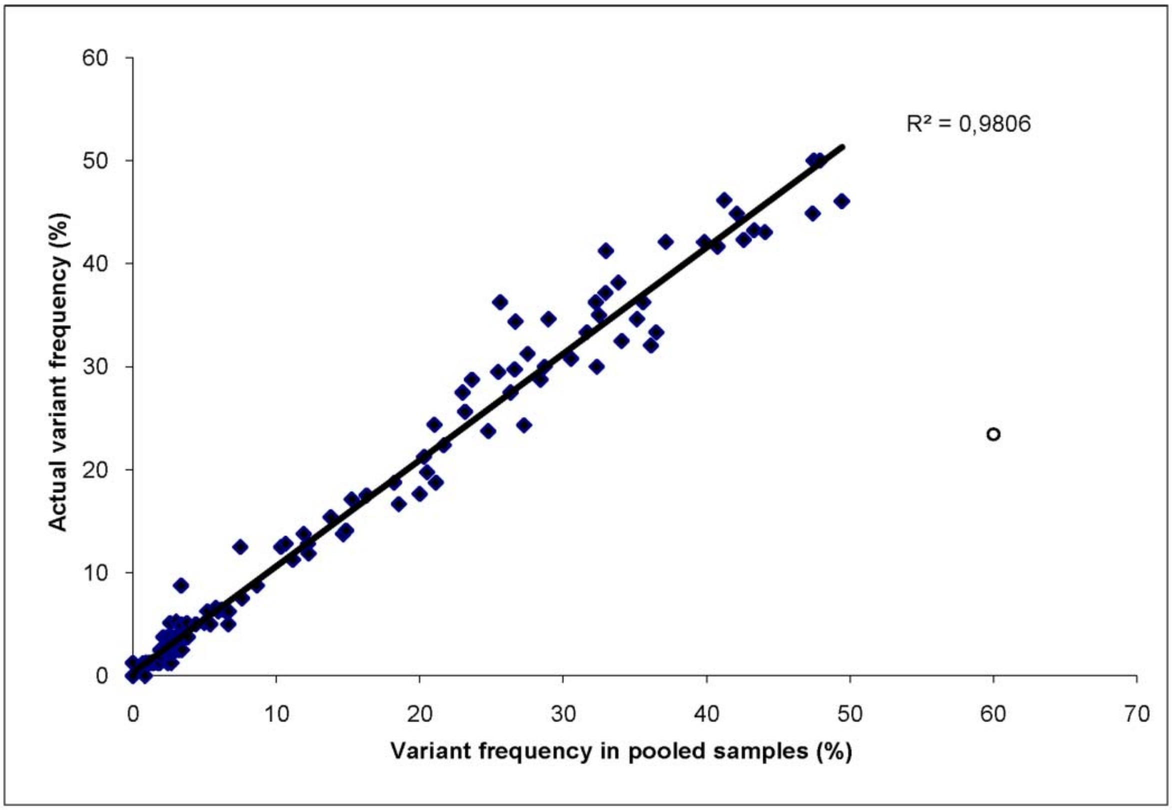

Supplement: Figure S2 — Allele frequencies estimated from pooled DNA samples (as determined by GS-FLX sequencing) versus the actual frequencies (as determined by genotyping the individual samples) in the different pools. °: outlier (corresponding to rs13398676, in SZ pool 1). (PDF) [file pone.0023450.s002.pdf]
