# Supplementary material for: Sequencing of DISC1 Pathway Genes Reveals Increased Burden of Rare Missense Variants in Schizophrenia Patients from a Northern Swedish Population
Source: PLoS One. 2011 Aug 11;6(8):e23450. doi: 10.1371/journal.pone.0023450 (PMC3154939; doi:10.1371/journal.pone.0023450)

Figure S3: Overview of the known interaction domains between the different proteins investigated, along with the positions of the variants identified in this study.

Protein lengths are given between brackets. Binding sites between two proteins are indicated along the line connecting them, with the binding site(s) on a certain protein closest to that protein (orange: binding sites on DISC1, blue: binding site on other proteins). The identified missense mutations are shown in a white area within the proteins' oval. Rare missense mutations (MAF ≤ 1%) are underlined, and mutations located in one of the binding sites are shown in italic. Note that the positions of many of the binding sites were not described/found in literature (indicated with '?').

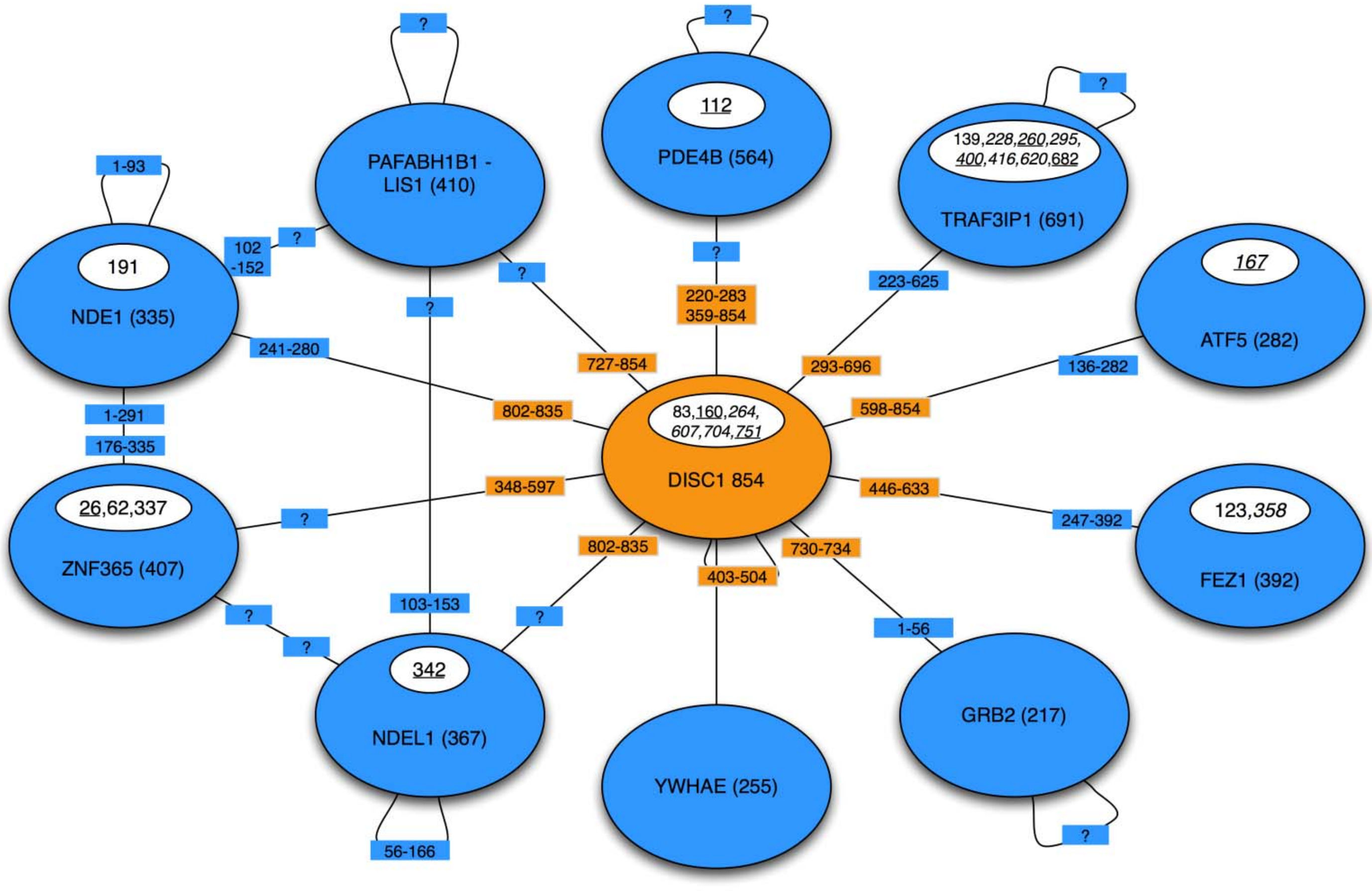

Supplement: Figure S3 — Overview of the known interaction domains between the different proteins investigated, along with the positions of the variants identified in this study. Protein lengths are given between brackets. Binding sites between two proteins are indicated along the line connecting them, with the binding site(s) on a certain protein closest to that protein (orange: binding sites on DISC1, blue: binding site on other proteins). The identified missense mutations are shown in a white area within the proteins' oval. Rare missense mutations (MAF ≤1%) are underlined, and mutations located in one of the binding sites are shown in italic. Note that the positions of many of the binding sites were not described/found in literature (indicated with ‘?’). (PDF) [file pone.0023450.s003.pdf]
