# Supplementary material for: Sequencing of DISC1 Pathway Genes Reveals Increased Burden of Rare Missense Variants in Schizophrenia Patients from a Northern Swedish Population
Source: PLoS One. 2011 Aug 11;6(8):e23450. doi: 10.1371/journal.pone.0023450 (PMC3154939; doi:10.1371/journal.pone.0023450)

Figure S4: Schematic representation of the overall domain architecture of each of the proteins investigated, highlighting the regions of predicted disorder (orange), along with regions having known homologous domains (blue). On each protein, the identified missense mutations are indicated. Mutations lying outside a disordered region are marked with an extra line.

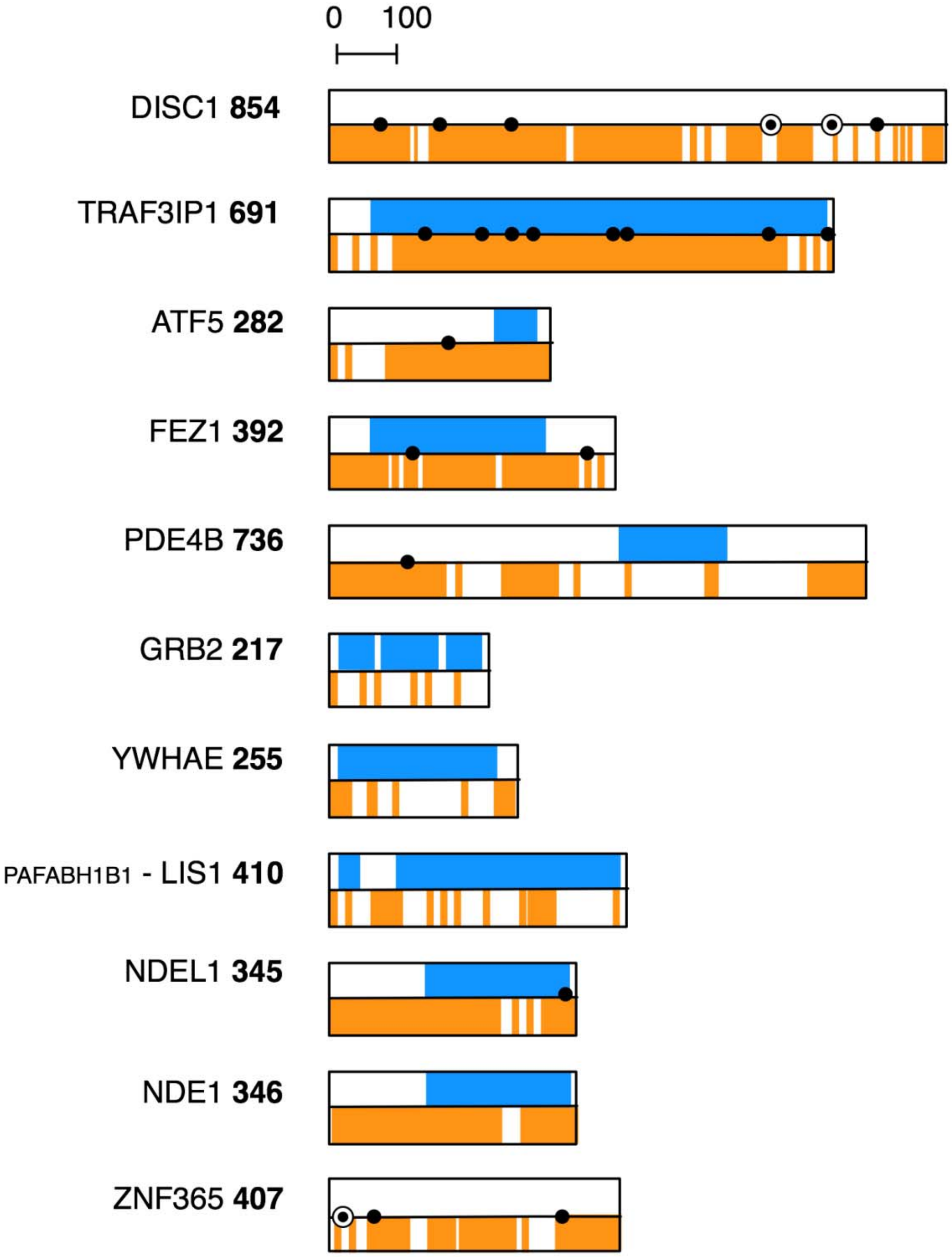

Supplement: Figure S4 — Schematic representation of the overall domain architecture of each of the proteins investigated, highlighting the regions of predicted disorder (orange), along with regions having known homologous domains (blue). On each protein, the identified missense mutations are indicated. Mutations lying outside a disordered region are marked with an extra line. (PDF) [file pone.0023450.s004.pdf]
