# Supplementary material for: Sequencing of DISC1 Pathway Genes Reveals Increased Burden of Rare Missense Variants in Schizophrenia Patients from a Northern Swedish Population
Source: PLoS One. 2011 Aug 11;6(8):e23450. doi: 10.1371/journal.pone.0023450 (PMC3154939; doi:10.1371/journal.pone.0023450)
Supplement: Table S1 — DISC1 interaction partners included in the study, and evidence for their involvement in psychiatric disease. (PDF) [file pone.0023450.s005.pdf]

Table S1: DISC1 interaction partners included in the study, and evidence for their involvement in psychiatric disease

Gene[a]	Location	Function	Evidence for involvement in psychiatric diseases	References [b]
DISC1	1q42.2	**"hub" protein** [c]	- genetic evidence (cytogenetics, linkage and association)	St.Clair et al.[1]; Blackwood et al.[2-3]; Ekelund et al[4-5]; Cannon et al.[6]; Callicott et al.[7]; Hennah et al.[8]; Hodgkinson et al.[9]; Saetre et al.[10]; Thomson et al.[11]; Kilpinen at el.[12]; Hashimoto et al.[13]
			- reduced expression in postmortem brain from SZ and UP patients, and in cell lines from BP patients	Sawamura et al.[14]; Maeda et al.[15]
			- impaired cognitive and behavioural phenotypes in humans and mutant mouse	Koike et al.[16]; Hikida et al.[17]; Clapcote et al.[18]; Pletnikov et al.[19]; Shen et al.[20]; Kvajo et al.[21]; Cannon et al.[6]; Burdick et al.[22]; Hennah et al.[23]; Li et al.[24]
ATF5 [25]	19q13.33	Signal transduction	- differential gene expression in lymphoblasts from MZ twins discordant for BP disorder	Kakiuchi et al.[26]
FEZ1 [27]	11q24.2	Neurite extension	- genetic evidence (association)	Yamada et al.[28]
			- reduced expression in postmortem brain from SZ patients	Lipska et al.[29]
			- physiological and behavioural effects in mutant mouse	
Grb2 [30]	17q24-q25	Nrg-EGF signaling	- no direct evidence, but interactions with multiple proteins associated with psychiatric illness, including DRD3, DRD4, PDE4D & ErbB receptors	Beard et al.[31]; Oldenhof et al.[32-33]; Corfas et al.[34]
NDE1 [35-36]	16p13	Neuronal migration & neurogenesis	- genetic evidence (linkage)	Hennah et al.[37]
NDEL1[25,36,38]	17p13.1	Neuronal migration & neurogenesis	- reduced expression in postmortem brain from SZ patients	Lipska et al.[29]; Tomppo et al.[39]
PAFAH1B1 [36]	17p13.3	Neuronal migration & neurogenesis	- reduced expression in postmortem brain from SZ patients	Lipska et al.[29]
PDE4B [40]	1p31.2	cAMP signaling	- genetic evidence (cytogenetics and association)	Millar et al.[40]; Tomppo et al.[39]; Pickard et al.[41]; Kähler et al.[42]
			- learning and memory alterations in Drosophila mutants	Davis[43]
			- behavioural effects in mouse mutants	O’Donnell and Zhang[44]; Siuciak et al.[45]; Zhang et al.[46]
			- altered gene expression upon antidepressant treatment	Takahashi et al.[47]; Miró et al.[48]; Dlaboga et al.[49]
TRAF3IP1 [25]	2q37.3	Signal transduction	- no direct evidence, but common interaction with DTNBP1, another major SZ risk gene	Camargo et al.[50]
YWHAE [51]	17p13.3	Signal transduction	- genetic evidence (association)	Ikeda et al.[52]
ZNF365 [53]	10q21.2	Neurite extension & cell division	- suggestive genetic evidence (linkage & association)	Liu et al.[54]; Marcheco-Teruel et al.[55]; Segurado et al.[56]

[a] References for interaction with DISC1 are given between brackets
[b] Data were reviewed in[57]
[c] As a hub protein, DISC1 is considered to be involved in all of the functions listed

REFERENCES

1. St Clair D, Blackwood D, Muir W, Carothers A, Walker M, et al. (1990) Association within a family of a balanced autosomal translocation with major mental illness. Lancet 336: 13-16.
2. Blackwood DH, Fordyce A, Walker MT, St Clair DM, Porteous DJ, et al. (2001) Schizophrenia and affective disorders--cosegregation with a translocation at chromosome 1q42 that directly disrupts brain-expressed genes: clinical and P300 findings in a family. Am J Hum Genet 69: 428-433.
3. Blackwood DH, Muir WJ (2004) Clinical phenotypes associated with DISC1, a candidate gene for schizophrenia. Neurotox Res 6: 35-41.
4. Ekelund J, Hennah W, Hiekkalinna T, Parker A, Meyer J, et al. (2004) Replication of 1q42 linkage in Finnish schizophrenia pedigrees. Mol Psychiatry 9: 1037-1041.
5. Ekelund J, Hovatta I, Parker A, Paunio T, Varilo T, et al. (2001) Chromosome 1 loci in Finnish schizophrenia families. Hum Mol Genet 10: 1611-1617.
6. Cannon TD, Hennah W, van Erp TG, Thompson PM, Lonnqvist J, et al. (2005) Association of DISC1/TRAX haplotypes with schizophrenia, reduced prefrontal gray matter, and impaired short- and long-term memory. Arch Gen Psychiatry 62: 1205-1213.
7. Callicott JH, Straub RE, Pezawas L, Egan MF, Mattay VS, et al. (2005) Variation in DISC1 affects hippocampal structure and function and increases risk for schizophrenia. Proc Natl Acad Sci U S A 102: 8627-8632.
8. Hennah W, Thomson P, McQuillin A, Bass N, Loukola A, et al. (2009) DISC1 association, heterogeneity and interplay in schizophrenia and bipolar disorder. Mol Psychiatry 14: 865-873.
9. Hodgkinson CA, Goldman D, Jaeger J, Persaud S, Kane JM, et al. (2004) Disrupted in schizophrenia 1 (DISC1): association with schizophrenia, schizoaffective disorder, and bipolar disorder. Am J Hum Genet 75: 862-872.
10. Saetre P, Agartz I, De Franciscis A, Lundmark P, Djurovic S, et al. (2008) Association between a disrupted-in-schizophrenia 1 (DISC1) single nucleotide polymorphism and schizophrenia in a combined Scandinavian case-control sample. Schizophr Res 106: 237-241.
11. Thomson PA, Harris SE, Starr JM, Whalley LJ, Porteous DJ, et al. (2005) Association between genotype at an exonic SNP in DISC1 and normal cognitive aging. Neurosci Lett 389: 41-45.
12. Kilpinen H, Ylisaukko-Oja T, Hennah W, Palo OM, Varilo T, et al. (2008) Association of DISC1 with autism and Asperger syndrome. Mol Psychiatry 13: 187-196.
13. Hashimoto R, Numakawa T, Ohnishi T, Kumamaru E, Yagasaki Y, et al. (2006) Impact of the DISC1 Ser704Cys polymorphism on risk for major depression, brain morphology and ERK signaling. Hum Mol Genet 15: 3024-3033.
14. Sawamura N, Sawamura-Yamamoto T, Ozeki Y, Ross CA, Sawa A (2005) A form of DISC1 enriched in nucleus: altered subcellular distribution in orbitofrontal cortex in psychosis and substance/alcohol abuse. Proc Natl Acad Sci U S A 102: 1187-1192.
15. Maeda K, Nwulia E, Chang J, Balkissoon R, Ishizuka K, et al. (2006) Differential expression of disrupted-in-schizophrenia (DISC1) in bipolar disorder. Biol Psychiatry 60: 929-935.
16. Koike H, Arguello PA, Kvajo M, Karayiorgou M, Gogos JA (2006) Disc1 is mutated in the 129S6/SvEv strain and modulates working memory in mice. Proc Natl Acad Sci U S A 103: 3693-3697.
17. Hikida T, Jaaro-Peled H, Seshadri S, Oishi K, Hookway C, et al. (2007) Dominant-negative DISC1 transgenic mice display schizophrenia-associated phenotypes detected by measures translatable to humans. Proc Natl Acad Sci U S A 104: 14501-14506.
18. Clapcote SJ, Lipina TV, Millar JK, Mackie S, Christie S, et al. (2007) Behavioral phenotypes of Disc1 missense mutations in mice. Neuron 54: 387-402.
19. Pletnikov MV, Ayhan Y, Nikolskaia O, Xu Y, Ovanesov MV, et al. (2008) Inducible expression of mutant human DISC1 in mice is associated with brain and behavioral abnormalities reminiscent of schizophrenia. Mol Psychiatry 13: 173-186, 115.
20. Shen S, Lang B, Nakamoto C, Zhang F, Pu J, et al. (2008) Schizophrenia-related neural and behavioral phenotypes in transgenic mice expressing truncated Disc1. J Neurosci 28: 10893-10904.
21. Kvajo M, McKellar H, Arguello PA, Drew LJ, Moore H, et al. (2008) A mutation in mouse Disc1 that models a schizophrenia risk allele leads to specific alterations in neuronal architecture and cognition. Proc Natl Acad Sci U S A 105: 7076-7081.
22. Burdick KE, Hodgkinson CA, Szeszko PR, Lencz T, Ekholm JM, et al. (2005) DISC1 and neurocognitive function in schizophrenia. Neuroreport 16: 1399-1402.
23. Hennah W, Tuulio-Henriksson A, Paunio T, Ekelund J, Varilo T, et al. (2005) A haplotype within the DISC1 gene is associated with visual memory functions in families with a high density of schizophrenia. Mol Psychiatry 10: 1097-1103.
24. Li W, Zhou Y, Jentsch JD, Brown RA, Tian X, et al. (2007) Specific developmental disruption of disrupted-in-schizophrenia-1 function results in schizophrenia-related phenotypes in mice. Proc Natl Acad Sci U S A 104: 18280-18285.
25. Morris JA, Kandpal G, Ma L, Austin CP (2003) DISC1 (Disrupted-In-Schizophrenia 1) is a centrosome-associated protein that interacts with MAP1A, MIPT3, ATF4/5 and NUDEL: regulation and loss of interaction with mutation. Hum Mol Genet 12: 1591-1608.
26. Kakiuchi C, Iwamoto K, Ishiwata M, Bundo M, Kasahara T, et al. (2003) Impaired feedback regulation of XBP1 as a genetic risk factor for bipolar disorder. Nat Genet 35: 171-175.
27. Miyoshi K, Honda A, Baba K, Taniguchi M, Oono K, et al. (2003) Disrupted-In-Schizophrenia 1, a candidate gene for schizophrenia, participates in neurite outgrowth. Mol Psychiatry 8: 685-694.
28. Yamada K, Nakamura K, Minabe Y, Iwayama-Shigeno Y, Takao H, et al. (2004) Association analysis of FEZ1 variants with schizophrenia in Japanese cohorts. Biol Psychiatry 56: 683-690.

29. Lipska BK, Peters T, Hyde TM, Halim N, Horowitz C, et al. (2006) Expression of DISC1 binding partners is reduced in schizophrenia and associated with DISC1 SNPs. Hum Mol Genet 15: 1245-1258.
30. Shinoda T, Taya S, Tsuboi D, Hikita T, Matsuzawa R, et al. (2007) DISC1 regulates neurotrophin-induced axon elongation via interaction with Grb2. J Neurosci 27: 4-14.
31. Beard MB, O'Connell JC, Bolger GB, Houslay MD (1999) The unique N-terminal domain of the cAMP phosphodiesterase PDE4D4 allows for interaction with specific SH3 domains. FEBS Lett 460: 173-177.
32. Oldenhof J, Ray A, Vickery R, Van Tol HH (2001) SH3 ligands in the dopamine D3 receptor. Cell Signal 13: 411-416.
33. Oldenhof J, Vickery R, Anafi M, Oak J, Ray A, et al. (1998) SH3 binding domains in the dopamine D4 receptor. Biochemistry 37: 15726-15736.
34. Corfas G, Roy K, Buxbaum JD (2004) Neuregulin 1-erbB signaling and the molecular/cellular basis of schizophrenia. Nat Neurosci 7: 575-580.
35. Millar JK, Christie S, Porteous DJ (2003) Yeast two-hybrid screens implicate DISC1 in brain development and function. Biochem Biophys Res Commun 311: 1019-1025.
36. Brandon NJ, Handford EJ, Schurov I, Rain JC, Pelling M, et al. (2004) Disrupted in Schizophrenia 1 and Nudel form a neurodevelopmentally regulated protein complex: implications for schizophrenia and other major neurological disorders. Mol Cell Neurosci 25: 42-55.
37. Hennah W, Tomppo L, Hiekkalinna T, Palo OM, Kilpinen H, et al. (2007) Families with the risk allele of DISC1 reveal a link between schizophrenia and another component of the same molecular pathway, NDE1. Hum Mol Genet 16: 453-462.
38. Ozeki Y, Tomoda T, Kleiderlein J, Kamiya A, Bord L, et al. (2003) Disrupted-in-Schizophrenia-1 (DISC-1): mutant truncation prevents binding to NudE-like (NUDEL) and inhibits neurite outgrowth. Proc Natl Acad Sci U S A 100: 289-294.
39. Tomppo L, Hennah W, Lahermo P, Loukola A, Ekelund J, et al. (2006) Association evidence from NUDEL and PDE4D support the DISC1-pathway concept in the etiology of schizophrenia. American Journal of Medical Genetics Part B: Neuropsychiatric Genetics 141B: 717.
40. Millar JK, Pickard BS, Mackie S, James R, Christie S, et al. (2005) DISC1 and PDE4B are interacting genetic factors in schizophrenia that regulate cAMP signaling. Science 310: 1187-1191.
41. Pickard BS, Thomson PA, Christoforou A, Evans KL, Morris SW, et al. (2007) The PDE4B gene confers sex-specific protection against schizophrenia. Psychiatr Genet 17: 129-133.
42. Kahler AK, Otnaess MK, Wirgenes KV, Hansen T, Jonsson EG, et al. (2010) Association study of PDE4B gene variants in Scandinavian schizophrenia and bipolar disorder multicenter case-control samples. Am J Med Genet B Neuropsychiatr Genet 153B: 86-96.
43. Davis RL (1996) Physiology and biochemistry of Drosophila learning mutants. Physiol Rev 76: 299-317.
44. O'Donnell JM, Zhang HT (2004) Antidepressant effects of inhibitors of cAMP phosphodiesterase (PDE4). Trends Pharmacol Sci 25: 158-163.
45. Siuciak JA, McCarthy SA, Chapin DS, Martin AN (2008) Behavioral and neurochemical characterization of mice deficient in the phosphodiesterase-4B (PDE4B) enzyme. Psychopharmacology (Berl) 197: 115-126.
46. Zhang HT, Huang Y, Masood A, Stolinski LR, Li Y, et al. (2008) Anxiogenic-like behavioral phenotype of mice deficient in phosphodiesterase 4B (PDE4B). Neuropsychopharmacology 33: 1611-1623.
47. Takahashi M, Terwilliger R, Lane C, Mezes PS, Conti M, et al. (1999) Chronic antidepressant administration increases the expression of cAMP-specific phosphodiesterase 4A and 4B isoforms. J Neurosci 19: 610-618.
48. Miro X, Perez-Torres S, Artigas F, Puigdomenech P, Palacios JM, et al. (2002) Regulation of cAMP phosphodiesterase mRNAs expression in rat brain by acute and chronic fluoxetine treatment. An in situ hybridization study. Neuropharmacology 43: 1148-1157.
49. Dlaboga D, Hajjhussein H, O'Donnell JM (2006) Regulation of phosphodiesterase-4 (PDE4) expression in mouse brain by repeated antidepressant treatment: comparison with rolipram. Brain Res 1096: 104-112.
50. Camargo LM, Collura V, Rain JC, Mizuguchi K, Hermjakob H, et al. (2007) Disrupted in Schizophrenia 1 Interactome: evidence for the close connectivity of risk genes and a potential synaptic basis for schizophrenia. Mol Psychiatry 12: 74-86.
51. Toyo-oka K, Shionoya A, Gambello MJ, Cardoso C, Leventer R, et al. (2003) 14-3-3epsilon is important for neuronal migration by binding to NUDEL: a molecular explanation for Miller-Dieker syndrome. Nat Genet 34: 274-285.
52. Ikeda M, Hikita T, Taya S, Uraguchi-Asaki J, Toyo-oka K, et al. (2008) Identification of YWHAE, a gene encoding 14-3-3epsilon, as a possible susceptibility gene for schizophrenia. Hum Mol Genet 17: 3212-3222.
53. Hattori T, Baba K, Matsuzaki S, Honda A, Miyoshi K, et al. (2007) A novel DISC1-interacting partner DISC1-Binding Zinc-finger protein: implication in the modulation of DISC1-dependent neurite outgrowth. Mol Psychiatry 12: 398-407.
54. Liu J, Juo SH, Dewan A, Grunn A, Tong X, et al. (2003) Evidence for a putative bipolar disorder locus on 2p13-16 and other potential loci on 4q31, 7q34, 8q13, 9q31, 10q21-24, 13q32, 14q21 and 17q11-12. Mol Psychiatry 8: 333-342.
55. Marcheco-Teruel B, Flint TJ, Wikman FP, Torralbas M, Gonzalez L, et al. (2006) A genome-wide linkage search for bipolar disorder susceptibility loci in a large and complex pedigree from the eastern part of Cuba. Am J Med Genet B Neuropsychiatr Genet 141B: 833-843.
56. Segurado R, Detera-Wadleigh SD, Levinson DF, Lewis CM, Gill M, et al. (2003) Genome scan meta-analysis of schizophrenia and bipolar disorder, part III: Bipolar disorder. Am J Hum Genet 73: 49-62.
57. Chubb JE, Bradshaw NJ, Soares DC, Porteous DJ, Millar JK (2008) The DISC locus in psychiatric illness. Mol Psychiatry 13: 36-64.
