# Supplementary material for: Sequencing of DISC1 Pathway Genes Reveals Increased Burden of Rare Missense Variants in Schizophrenia Patients from a Northern Swedish Population
Source: PLoS One. 2011 Aug 11;6(8):e23450. doi: 10.1371/journal.pone.0023450 (PMC3154939; doi:10.1371/journal.pone.0023450)
Supplement: Table S2 — Allele frequencies of pooled DNA and individual samples, as determined by pyrosequencing. (PDF) [file pone.0023450.s006.pdf]

Table S2: Allele frequencies of pooled DNA and individual samples, as determined by pyrosequencing

	rs67705083		rs778294		rs1341402	
	% G	% T	% C	% T	% A	% G
Control samples N° 1-40	64.91	35.10	73.58	26.42	73.84	26.16
Control pool 1[a]	61.28 ± 0.50	38.73 ± 0.50	72.45 ± 1.68	27.55 ± 1.68	69.95 ± 4.27	30.05 ± 4.27
Control samples N° 41-80	66.76	33.24	72.01	27.99	74.97	25.03
Control pool 2 [a]	63.05 ± 1.41	34.45 ± 1.41	72.93 ± 1.47	27.08 ± 1.47	74.43 ± 0.74	25.58 ± 0.74
Patient samples N° 1-40	60.06	39.94	72.29	27.71	73.88	26.12
Patient pool 1 [a]	59.23 ± 1.48	40.78 ± 1.48	69.65 ± 1.45	30.35 ± 1.45	71.9 ± 0.80	28.1 ± 0.80
Patient samples N° 41-80	64.43	35.58	76.90	23.10	73.94	26.06
Patient pool 2 [a]	61.43 ± 0.50	38.58 ± 0.50	78.73 ± 1.77	21.28 ± 1.77	73.35 ± 0.75	26.65 ± 0.75

[a] Average allele frequencies of 4 replicates (± SD)
