# Supplementary material for: Sequencing of DISC1 Pathway Genes Reveals Increased Burden of Rare Missense Variants in Schizophrenia Patients from a Northern Swedish Population
Source: PLoS One. 2011 Aug 11;6(8):e23450. doi: 10.1371/journal.pone.0023450 (PMC3154939; doi:10.1371/journal.pone.0023450)
Supplement: Table S3 — Properties of amplicon subset selected for false negative rate estimation using Sanger sequencing. (PDF) [file pone.0023450.s007.pdf]

Table S3: Properties of amplicon subset selected for false negative rate estimation using Sanger sequencing

Property	Full amplicon set	Amplicon subset
# amplicons	153	14
Distribution of variants/amplicon [b]		
# amplicons without variants	95 (0.62) [a]	9 (0.64) [a]
# amplicons with variants	58 (0.38) [a]	5 (0.36) [a]
# variants [b]	77 (0.50) [a]	7 (0.50) [a]
# amplicons with low read count [c]	10 (0.065) [a]	1 (0.07) [a]
Average read count	1505	1308
# screened bases	33864	3111

As the subsample was only sequenced in patients, data are shown for the patient pools only (excluding control-specific variants)

[a] Absolute number (relative to the number of amplicons)

[b] Includes both coding and non-coding variants (quality score ≥20)

[c] < 500 reads/amplicon in both patient pools (i.e. <12.5 reads/individual)
