# Supplementary material for: Sequencing of DISC1 Pathway Genes Reveals Increased Burden of Rare Missense Variants in Schizophrenia Patients from a Northern Swedish Population
Source: PLoS One. 2011 Aug 11;6(8):e23450. doi: 10.1371/journal.pone.0023450 (PMC3154939; doi:10.1371/journal.pone.0023450)
Supplement: Table S4 — Mutation burden of identified rare nonsynonymous mutations (MAF<1%), stratified by gene. (PDF) [file pone.0023450.s008.pdf]

Table S4: Mutation burden of identified rare nonsynonymous mutations (MAF<1%), stratified by gene

Gene	Variants	Av. # variant alleles/individual[a]		SZ/co ratio[b]	P[c]	# bp sequenced	Mutation burden /1000 bp[d]
		co	SZ				
ATF5	R167C	0.004	0.010	2.50	0.211	955	0.010
DISC1	W160L, E751Q	0.021	0.041	1.95	0.112	5198	0.008
NDEL1	P342S	0.004	0.000	0.00	0.500	2156	0.000
PDE4B	A112G	0.012	0.021	1.75	0.264	5477	0.004
TRAF3IP1	D400A, E260K, V682X	0.004	0.008	2.00	0.342	4905	0.002
ZNF365	P26L	0.002	0.006	3.00	0.244	4360	0.001
	**Combined**	**0.047**	**0.086**	**1.85**	**0.015**		

Abbreviations: co, control individuals; SZ, schizophrenia patients
Significant values (P<0.05) are shown in bold
[a] Mutation burden, defined as the average number of variant alleles/person
[b] Fold increase of mutation burden in patients versus control individuals
[c] Empirical P-values, obtained by performing 5000 permutations
[d] Mutation burden in patients, relative to the number of bases sequenced for each gene
