# Supplementary material for: Sequencing of DISC1 Pathway Genes Reveals Increased Burden of Rare Missense Variants in Schizophrenia Patients from a Northern Swedish Population
Source: PLoS One. 2011 Aug 11;6(8):e23450. doi: 10.1371/journal.pone.0023450 (PMC3154939; doi:10.1371/journal.pone.0023450)
Supplement: Text S1 — Supplementary Materials and Methods, Results and Discussion. (PDF) [file pone.0023450.s009.pdf]

MATERIALS AND METHODS

Patient ascertainment and assessment

The ascertainment and assessment procedure of the patients consisted of several phases. We used several sources of data, known to yield sufficient reliability and validity of a DSM-IV diagnosis of schizophrenia. The patients were initially identified through the in-patient hospital registers, and all had at least on one occasion received a discharge diagnosis of SZ. The DSM- IV discharge diagnosis of SZ showed a very high agreement with the DSM-IV research diagnosis based on analyses of records, collateral information and semi-structured interviews [1], findings that support that Swedish psychiatric registers are an excellent source of information with regard to diagnoses within the schizophrenic spectra.

The ascertainment of the patients was performed between 1992 and 2005. Clinical characterization was assessed by trained research nurses and research psychiatrists by the use of register data, data from psychiatric records, collateral information from relatives and semi-structured interviews, the Diagnostic Interview for Genetic Studies-DIGS [2], the Family Interview for Genetic Studies-FIGS and the Schedules for Clinical Assessment in Neuropsychiatry- SCAN [3], an approach that yields a high reliability and validity of a DSM-IV diagnosis of schizophrenia [1]. The final diagnosis was determined by the consensus of 2 research psychiatrists and only patients for whom full consensus was reached were included in the study.

DNA quantification and pyrosequencing of individual and pooled DNA samples

The DNA samples to be pooled were first diluted in TE (10mM Tris and 1mM EDTA, pH 7.5) to a concentration of ~0.5-1 ng/µl, and an 11-point dilution series (from 2 to zero ng/µl) was prepared from commercial human genomic DNA (Roche Applied Science, Penzberg, Germany) as a standard. Immediately before quantification PicoGreen (Molecular Probes, Invitrogen, Paisly, UK) was diluted 200 times in TE, and mixed with the samples in a 1:1 ratio. Fluorescent detection was done using a FL600 microplate fluorescence reader (Bio-Tek, Winooski, VT, USA). The fluorescence values of the standard series were used to generate a calibration curve, and finally the concentration of each test sample was calculated based on the resulting equation.

Following DNA pooling, three pyrosequencing assays (for SNPs rs67705083, rs778294 and rs1341402) were carried out, to control the efficiency of the pooling procedure. For each of the DNA samples and pools (4 replicates/pool), PCR reactions were carried out in a volume of 20 µL, consisting of 10 ng DNA, 1x Titanium Taq PCR buffer (Clontech, Palo Alto, CA), 0.25 µM biotin-labeled primers, 0.35µM primer 2, 0.1µL Titanium Taq polymerase (Clontech) and 0.15 mM of each dNTP (Invitrogen). PCR conditions were 3’ at 95°C, 35 cycles of 30” at 95°C, 30” at 60°C and 45” at 72°C, followed by a final extension of 5’ at 72°C. Pyrosequencing reactions were performed according to the instructions of the manufacturer (Biotage, Uppsala, Sweden). In short, biotinylated PCR products were immobilized onto streptavidin-coated sepharose beads (Amersham Biosciences, Uppsala, Sweden). Biotinylated ssDNA was obtained by incubating the immobilized PCR products in 0.5 M NaOH, followed by two sequential washes in 10 mM Tris-Acetate (pH 7.6). Primer annealing was performed by incubation for 2 min at 80°C, followed by 5 min at room temperature. Finally, annealed templates were loaded on a PSQ HS96 pyrosequencer and analyzed using the PSQ HS96 SNP Software (allele quantification mode) (Biotage). For each pool, the allele frequencies of the different SNPs were determined and compared to the frequencies in the individual DNA samples constituting the pools.

For all 3 SNP assays, the allele frequencies of the 4 DNA pools corresponded very well to the frequencies obtained with the individual samples (**Table S2**).

Multiplex PCR design and reaction conditions

An optimal set of targets was defined to cover all coding exons and splice junctions of the selected target genes. Multiplex primer design was done as described earlier[4] based on the following sequences: NM_012068 (*ATF5*); NM_018662, NM_001012959, NM_001012958, NM_001012957 (*DISC1*); NM_005103, NM_022549 (*FEZ1*); NM_203506, NM_002086 (*Grb2*); NM_017668 (*NDE1*); NM_030808, NM_001025579 (*NDEL1*); NM_000430 (*PAFAH1B1*); NM_001037341, NM_001037339, NM_001037340, NM_002600 (*PDE4B*); NM_015650 (*TRAF3IP1*); NM_006761 (*YWHAE*); NM_014951, NM_199452, NM_199451, NM_199450 (*ZNF365*) (NCBI Build 36). Each primer was provided with one of the 454 adaptors sequences[5], to enable subsequent GS-FLX based sequencing. The resulting primers were ordered from Integrated DNA Technologies (Leuven, Belgium) and first tested in simplex PCR reactions, using 10 ng genomic DNA and 10 pmol per primer. Reactions were carried out in a volume of 25 µL, containing 1x Titanium Taq PCR buffer (Clontech), 0.125 µL Titanium Taq polymerase (Clontech), and 0.25 mM of each dNTP (Invitrogen). PCR cycle conditions were 2’ at 98°C, 30 cycles of 45” at 95°C, 45” at 60°C, and 2’ at 68°C, and a final extension step of 10’ at 72°C. Multiplex reactions were performed

using the same reaction conditions, but starting from 100 ng pooled genomic DNA. Primer concentrations were optimized to achieve an equal amplification of each target, and varied from 0.032 pmol/µL to 0.28 pmol/µL final concentration.

Variant detection and validation

Identified potential variants were validated in the complete association sample using the MassARRAY based genotyping, following the protocol provided by Sequenom (Hamburg, Germany). PCR and extension primers were designed using the Assay Design 3.0 program (Sequenom). Briefly, 20 ng genomic DNA was PCR amplified using Titanium Taq DNA Polymerase (Clontech). PCR primers were used at 200 nM final concentrations for a PCR volume of 10 µL. PCR conditions were 15' at 95°C, 45 cycles of 20" at 94°C, 30" at 56°C, and 1' at 72°C, followed by a final extension at of 3' at 72°C. PCR products were treated with shrimp alkaline phosphatase (Sequenom) for 20 min at 37°C to remove excess dNTPs, and finally, ThermoSequenase (Sequenom) was used for the base extension reactions. Analysis and scoring were performed using the program Typer 3.3 (Sequenom).
Sanger sequencing was performed using 10 ng of genomic DNA with 10 pmol of each primer in a standard PCR reaction, followed by ExoSAPit treatment (Amersham Biosciences) and subsequently sequenced using the Big Dye terminator cycle sequencing kit v3.1 according to the manufacturer's instructions (Applied Biosystems, Foster City, CA). Sequencing reactions were run on a 3730XL DNA Analyzer (Applied Biosystems) and the resulting trace files were analyzed using NovoSNPv3.0[6].

***In silico* functional analyses**

For each of the variants, nucleotide conservation was calculated using the GERP (Genome Evolutionary Rate Profiling) score[7]. This score reflects the extent of divergence at a specific base position among mammals, with negative values corresponding to neutral or unconstrained sites and positive values corresponding to constrained or slowly evolving sites.
Effects on potential splice sites and *cis*-acting elements (exonic splice enhancers (ESE) and silencers (ESS)) were investigated using Human Splicing Finder v2.4 (http://www.umd.be/HSF)[8], an online bioinformatics tool that combines all available matrices for auxiliary sequence prediction, including the Shapiro-Senapathy matrix[9] and MaxEnt modeling[10] for predicting splice donor/acceptor motifs, and RESCUE-ESE[11], ESE-Finder[12], PESX[13] and HSF[8] for predicting splicing enhancers/silencers. For the prediction of *cis*-acting elements, only mutations located within 30 nt of the nearest exon-intron boundary were taken into account, as suggested previously[14].
The missense coding variants were functionally and structurally characterized using SNPeffect[15]. The sequence variants were checked for putative changes in aggregation tendency, chaperone binding, post translational modifications (acetylation, glycosylation and phosphorylation), cellular processing (degradation rate and targeting signals), attachment of lipid anchors, and whenever experimental structures of homologous proteins were available 3D structural models were built using the Yasara toolsuite[16]. These models where subsequently used to evaluate the difference in protein stability introduced by the amino acid substitution using the FoldX algorithm[17]. Sequences where also examined for intrinsically disordered regions using the DisProt predictor[18], and disorder content was compared to (i) the human proteome (i.e. 20320 sequences, as downloaded from the UniProt protein resource), (ii) a set of brain proteins (7160 sequences, taken from GeneAtlas[19]) and (iii) a set of schizophrenia candidate proteins (670 sequences, taken from the Schizophrenia Gene Resource, http://bioinfo.mc.vanderbilt.edu/SZGR/). Differences in disorder content between the DISC proteins and these 3 reference datasets were investigated by a Mann-Whitney test. In addition, evolutionary amino acid conservation was analyzed using SIFT (Sorting Intolerant From Tolerant; http://sift.jcvi.org)[20], in combination with PolyPhen Polymorphism Phenotyping; http://genetics.bwh.harvard.edu/pph)[21] and Panther (http://www.pantherdb.org)[22-23]. All three programs use sequence homology among related genes and domains across species to predict whether an amino acid substitution affects protein function. The PolyPhen algorithm in addition also uses structural information. Only variants concordantly scored as damaging by at least two algorithms were considered as potentially functional.
Finally, 5' UTR variants were analyzed for predicted transcription factor binding sites (TFBSs), and potential disruption of miRNA target sites. TFBSs in the promotor region of the candidate genes were identified by scanning the region 5kb upstream of the genes with the Match program[24] from the Transfac database[25]. As a complementary approach, we also used MatScan and JASPARS[26] collection of matrices to search for TFBSs. Next, we identified the TFBSs conserved between species (human and mouse) using meta-alignments[27]. SNPs mapped into these conserved TFBSs are considered to affect the expression of the corresponding gene.

For the prediction of human miRNA target sites in the 3' UTR of the candidate genes, we used the miRanda algorithm[28]. This algorithm uses dynamic programming to search for maximal local complementarity alignments, which correspond to a double-stranded anti-parallel duplex. P-values for the detected target sites were calculated based on the statistical model proposed by Rehmsmeier and colleagues[29].

RESULTS

454 sequencing validation and variant discovery
454 sequence analysis was performed on the 4 DNA pools and on 1 individual patient DNA sample. Sequencing of the individual DNA sample resulted in a total of 610 kb sequence information, corresponding to ~3,200 reads with an average length of 190 bp. Sequencing of the pooled DNA samples generated ~95.4 Mb, corresponding to ~432,000 reads (average length 220 bp) for the patient pools, and ~97.2 Mb, corresponding to ~448,000 reads (average length 217 bp) for the control pools. Respectively, 96% and 93.5% of these reads could be mapped to the reference sequence, indicating a high PCR amplification specificity. The average number of mapped reads per amplicon was comparable between patient and control pools (1356 versus 1368 reads/amplicon, respectively (~34 reads/individual)), justifying a comparison between these two groups.
The number of reads is homogeneously distributed over the different amplicon pools, except for multiplex reaction 12, where a considerably lower number of reads was obtained (**Figure S1**). More specifically, the average observed read count of > 95% of the amplicons falls within a 10-fold range, and when we exclude the 8 amplicons with the lowest average read count, the observed read counts of >95% of the remaining amplicons fall within a narrow 5-fold range, demonstrating the uniformity of amplification.

Assessment of false negative rate
To estimate the incidence of false negatives in our sequencing dataset, we subjected a representative subset of amplicons to traditional Sanger-based sequencing. These amplicons were selected such that they were comparable to the full set of amplicons according to the criteria described in **Table S3**. In short, 14 amplicons (~9.2% of all amplicons) were Sanger sequenced in the 80 patient samples. This resulted in the identification of 1 extra variant in an amplicon with a low 454 read count, indicating that the occurrence of type II errors was negligible as long as sequence coverage was sufficient (≥500 reads/amplicon).

Analysis of pooling efficiency
We evaluated the performance of the pooled sequencing strategy for variant discovery by generating independent 454 sequencing data from an individual DNA sample that was also present in one of the patient pools. In this individual DNA sample, 20 variants were identified (indicated with an asterisk in Table 1). All 20 variants were also detected in the corresponding patient pool showing that the pooling of the DNA samples, and subsequent multiplex PCR and sequencing steps, had been successful.
Next, we evaluated the accuracy of our sample pooling approach and its value for SNP frequency estimation, by comparing the minor allele frequencies of the variants in the pooled samples (as determined by GS-FLX sequencing) with their actual frequencies (as determined by genotyping), for each of the validated variants (**Figure S2**). Except for 1 outlier (detected in an amplicon with only 5 reads), the observed and predicted frequencies correlated very well (R^2=0.98) across a wide range of frequencies – varying from a single allele (1.25%) to the maximum allele frequency of 50%. These findings not only demonstrate the accuracy of our DNA sample pooling, but also indicate the usefulness of this approach for estimating the population frequency of both rare and common alleles.

DISCUSSION

Achieving adequate coverage is one of the most important factors in the design of a (multiplexed) targeted sequencing experiment[30]. As singleton variants will theoretically have a frequency of 1.25% in a pool of 40 samples, 1000x coverage per amplicon (*i.e.* 12.5 reads/allele) should be sufficient to resolve all variants by at least 10 reads. With an average read count of 34 reads per individual (17 per allele), our sequencing approach resulted in an overall coverage amply sufficient to detect singletons. Furthermore, the uniform distribution of the read counts over the different amplicons (Figure S1), and the equal number of reads between patients and controls provided a good basis for polymorphism discovery and comparison between these two populations. However, 10 amplicons – mostly belonging to multiplex reaction 12 – showed a consistently lower number of reads (i.e. <500 reads, corresponding to <7 reads/allele) in patients and controls. This might partially be caused by a higher GC content of these amplicons compared to the other targets (mean GC% = 64.6 ± 14.2 versus 46.4 ± 9.4).

By using stringent criteria for SNP discovery, we found that ~82% of the discovered variants (50 out of 61) were true positives. This relatively high false positive rate can be explained by the experimental errors introduced by DNA amplification and 454 sequencing. Because of this, substitutions observed at low frequencies will be a mixture of technical errors and true mutations, and sequencing data need to be filtered to be able to distinguish true signal from noise. Assuming a GS-FLX error rate of ~0.1 -0.25%[31-32] and a total of ~200Mb mapped nucleotides, we expect at least $2x10^5$ errors. After filtering the detected substitutions (based on flow quality and frequency of variant reads), we succeeded to drastically reduce this number of false positive errors, demonstrating the specificity of our SNP calling algorithm. Sequencing a representative subset of amplicons for false negative rate determination showed that the occurrence of type II errors was negligible as long as sequence coverage was sufficient (≥500 reads/amplicon). This observation is in line with two previous reports, describing multiplexed bar-coded resequencing[29] and simplex PCR-based pooled-sample sequencing[33], that also emphasized the importance of coverage for controlling false negative rates. However, as the frequency threshold for variant detection in our discovery set (≥0.8%) was quite close to the expected frequency of a singleton mutation (1.25%), it is still possible that a few (rare) mutations were missed. More individuals will need to be sequenced to resolve this. Eighteen of the 50 validated variants had a MAF <1%, demonstrating the sensitivity of our pooled-sample sequencing approach to detect rare variants. Evidently, the discovery of rare variants also depends on the number of individuals that are screened; even if all variants in a sample are successfully identified (i.e. no false negatives), the probability of observing a variant with a population frequency of 0.1% in a sample of 160 individuals, is only 27% (i.e. $[1-(1-p)^{2N}]$ with p= minor allele frequency and N= number of individuals sampled). If the sample size is increased to 1000 genomes, the proportion of variants discovered increases to 86%. In this study, we sequenced 160 individuals (80 patients and 80 controls), allowing the discovery of a reasonable proportion (80%) of all variants with a frequency of 0.5% present in the population from which our sample was ascertained, and 96% of all 1% frequency variants. Furthermore, by using a screening sample enriched for patients with an early onset age, we could further improve the power of mutation discovery, as this subgroup of patients might have a stronger genetic burden for the illness [34-36].

As one of the performance measures of our experimental platform, we evaluated whether the pooled-sample sequencing strategy also allows to accurately estimate the frequencies of the detected variants. Indeed, not only would variant discovery be considerably accelerated by using pooled samples, this method could also be used to determine the mutation spectrum associated with a certain phenotype of interest, or for combined variant discovery and association analyses – provided the population frequencies of both rare and common alleles can accurately be quantified. In line with previous studies [33,37], our results (Figure S2) indicate that massive parallel sequencing of pooled samples is a highly valuable approach enabling both the discovery of (rare and common) alleles and a simultaneous quantification of the identified mutations.

Both mutation burden and individual variant analyses were performed on the complete association sample (i.e. including the discovery samples). Contrary to the use of patient samples *only* for variant discovery, inclusion of an equally large control sample in the discovery phase, does not lead to an inflation of type I errors [38]. To show that the inclusion of our discovery samples does not lead to type I error inflation, we calculated the expected number of variant alleles per individual, for each of the detected variants, in the two (complete) populations. These calculations were based on the variant's MAF, and took into account whether or not the variant was *a priori* detected in that population [38]. We found the number of expected variant alleles per individual to be equal in patients and controls, across the different mutation types and variant frequencies investigated (P> 0.86), thereby justifying the inclusion of our discovery sample for association analyses.

REFERENCES

1. Ekholm B, Ekholm A, Adolfsson R, Vares M, Osby U, et al. (2005) Evaluation of diagnostic procedures in Swedish patients with schizophrenia and related psychoses. Nord J Psychiatry 59: 457-464.
2. Nurnberger JI, Jr., Blehar MC, Kaufmann CA, York-Cooler C, Simpson SG, et al. (1994) Diagnostic interview for genetic studies. Rationale, unique features, and training. NIMH Genetics Initiative. Arch Gen Psychiatry 51: 849-859; discussion 863-844.
3. Wing JK, Babor T, Brugha T, Burke J, Cooper JE, et al. (1990) SCAN. Schedules for Clinical Assessment in Neuropsychiatry. Arch Gen Psychiatry 47: 589-593.
4. Goossens D, Moens LN, Nelis E, Lenaerts AS, Glassee W, et al. (2009) Simultaneous mutation and copy number variation (CNV) detection by multiplex PCR-based GS-FLX sequencing. Hum Mutat 30: 472-476.
5. Margulies M, Egholm M, Altman WE, Attiya S, Bader JS, et al. (2005) Genome sequencing in microfabricated high-density picolitre reactors. Nature 437: 376-380.
6. Weckx S, Del-Favero J, Rademakers R, Claes L, Cruts M, et al. (2005) novoSNP, a novel computational tool for sequence variation discovery. Genome Res 15: 436-442.
7. Cooper GM, Stone EA, Asimenos G, Green ED, Batzoglou S, et al. (2005) Distribution and intensity of constraint in mammalian genomic sequence. Genome Res 15: 901-913.
8. Desmet FO, Hamroun D, Lalande M, Collod-Beroud G, Claustres M, et al. (2009) Human Splicing Finder: an online bioinformatics tool to predict splicing signals. Nucleic Acids Res 37: e67.
9. Shapiro MB, Senapathy P (1987) RNA splice junctions of different classes of eukaryotes: sequence statistics and functional implications in gene expression. Nucleic Acids Res 15: 7155-7174.
10. Yeo G, Burge CB (2004) Maximum entropy modeling of short sequence motifs with applications to RNA splicing signals. J Comput Biol 11: 377-394.
11. Fairbrother WG, Yeo GW, Yeh R, Goldstein P, Mawson M, et al. (2004) RESCUE-ESE identifies candidate exonic splicing enhancers in vertebrate exons. Nucleic Acids Res 32: W187-190.
12. Cartegni L, Wang J, Zhu Z, Zhang MQ, Krainer AR (2003) ESEfinder: A web resource to identify exonic splicing enhancers. Nucleic Acids Res 31: 3568-3571.
13. Zhang XH, Chasin LA (2004) Computational definition of sequence motifs governing constitutive exon splicing. Genes Dev 18: 1241-1250.
14. Fairbrother WG, Holste D, Burge CB, Sharp PA (2004) Single nucleotide polymorphism-based validation of exonic splicing enhancers. PLoS Biol 2: E268.
15. Reumers J, Conde L, Medina I, Maurer-Stroh S, Van Durme J, et al. (2008) Joint annotation of coding and non-coding single nucleotide polymorphisms and mutations in the SNPeffect and PupaSuite databases. Nucleic Acids Res 36: D825-829.
16. Krieger E, Koraimann G, Vriend G (2002) Increasing the precision of comparative models with YASARA NOVA--a self-parameterizing force field. Proteins 47: 393-402.
17. Schymkowitz J, Borg J, Stricher F, Nys R, Rousseau F, et al. (2005) The FoldX web server: an online force field. Nucleic Acids Res 33: W382-388.
18. Peng K, Radivojac P, Vucetic S, Dunker AK, Obradovic Z (2006) Length-dependent prediction of protein intrinsic disorder. BMC Bioinformatics 7: 208.
19. Su AI, Wiltshire T, Batalov S, Lapp H, Ching KA, et al. (2004) A gene atlas of the mouse and human protein-encoding transcriptomes. Proc Natl Acad Sci U S A 101: 6062-6067.
20. Ng PC, Henikoff S (2001) Predicting deleterious amino acid substitutions. Genome Res 11: 863-874.
21. Ramensky V, Bork P, Sunyaev S (2002) Human non-synonymous SNPs: server and survey. Nucleic Acids Res 30: 3894-3900.
22. Thomas PD, Campbell MJ, Kejariwal A, Mi H, Karlak B, et al. (2003) PANTHER: a library of protein families and subfamilies indexed by function. Genome Res 13: 2129-2141.
23. Thomas PD, Kejariwal A, Guo N, Mi H, Campbell MJ, et al. (2006) Applications for protein sequence-function evolution data: mRNA/protein expression analysis and coding SNP scoring tools. Nucleic Acids Res 34: W645-650.
24. Kel AE, Gossling E, Reuter I, Cheremushkin E, Kel-Margoulis OV, et al. (2003) MATCH: A tool for searching transcription factor binding sites in DNA sequences. Nucleic Acids Res 31: 3576-3579.
25. Wingender E, Chen X, Hehl R, Karas H, Liebich I, et al. (2000) TRANSFAC: an integrated system for gene expression regulation. Nucleic Acids Res 28: 316-319.
26. Vlieghe D, Sandelin A, De Bleser PJ, Vleminckx K, Wasserman WW, et al. (2006) A new generation of JASPAR, the open-access repository for transcription factor binding site profiles. Nucleic Acids Res 34: D95-97.

27. Blanco E, Messeguer X, Smith TF, Guigo R (2006) Transcription factor map alignment of promoter regions. PLoS Comput Biol 2: e49.
28. Enright AJ, John B, Gaul U, Tuschl T, Sander C, et al. (2003) MicroRNA targets in Drosophila. Genome Biol 5: R1.
29. Rehmsmeier M, Steffen P, Hochsmann M, Giegerich R (2004) Fast and effective prediction of microRNA/target duplexes. RNA 10: 1507-1517.
30. Craig DW, Pearson JV, Szelinger S, Sekar A, Redman M, et al. (2008) Identification of genetic variants using bar-coded multiplexed sequencing. Nat Methods 5: 887-893.
31. Solmone M, Vincenti D, Prosperi MC, Bruselles A, Ippolito G, et al. (2009) Use of massively parallel ultradeep pyrosequencing to characterize the genetic diversity of hepatitis B virus in drug-resistant and drug-naive patients and to detect minor variants in reverse transcriptase and hepatitis B S antigen. J Virol 83: 1718-1726.
32. De Grassi A, Segala C, Iannelli F, Volorio S, Bertario L, et al. (2010) Ultradeep sequencing of a human ultraconserved region reveals somatic and constitutional genomic instability. PLoS Biol 8: e1000275.
33. Ingman M, Gyllensten U (2009) SNP frequency estimation using massively parallel sequencing of pooled DNA. Eur J Hum Genet 17: 383-386.
34. DeLisi LE, Bass N, Boccio A, Shields G, Morganti C (1994) Age of onset in familial schizophrenia. Arch Gen Psychiatry 51: 334-335.
35. Albus M, Maier W (1995) Lack of gender differences in age at onset in familial schizophrenia. Schizophr Res 18: 51-57.
36. Kumra S, Charles Schulz S (2008) Editorial: research progress in early-onset schizophrenia. Schizophr Bull 34: 15-17.
37. Druley TE, Vallania FL, Wegner DJ, Varley KE, Knowles OL, et al. (2009) Quantification of rare allelic variants from pooled genomic DNA. Nat Methods 6: 263-265.
38. Li B, Leal SM (2009) Discovery of rare variants via sequencing: implications for the design of complex trait association studies. PLoS Genet 5: e1000481.
